# Comprehensive review and updated analysis of DNA methylation in hepatocellular carcinoma: From basic research to clinical application

**DOI:** 10.1002/ctm2.70066

**Published:** 2024-10-27

**Authors:** Lin Su, Jiawen Bu, Jiahui Yu, Mila Jin, Guanliang Meng, Xudong Zhu

**Affiliations:** ^1^ Department of Pain Management Shengjing Hospital of China Medical University Shenyang Liaoning China; ^2^ Department of Colorectal Surgery Shengjing Hospital of China Medical University Shenyang Liaoning China; ^3^ Department of Ultrasound Shengjing Hospital of China Medical University Shenyang Liaoning China; ^4^ Department of Operation Room The First Hospital of China Medical University Shenyang Liaoning China; ^5^ Department of Urology Shengjing Hospital of China Medical University Shenyang Liaoning China; ^6^ Department of Oncology Shengjing Hospital of China Medical University Shenyang Liaoning China; ^7^ Department of General Surgery Cancer Hospital of China Medical University Shenyang Liaoning China

**Keywords:** DNA methylation, DNA methyltransferase, hepatocellular carcinoma, inhibitors, malignant progression, sorafenib resistance

## Abstract

Hepatocellular carcinoma (HCC) is a primary malignant tumour, ranking second in global mortality rates and posing significant health threats. Epigenetic alterations, particularly DNA methylation, have emerged as pivotal factors associated with HCC diagnosis, therapy, prognosis and malignant progression. However, a comprehensive analysis of the DNA methylation mechanism driving HCC progression and its potential as a therapeutic biomarker remains lacking. This review attempts to comprehensively summarise various aspects of DNA methylation, such as its mechanism, detection methods and biomarkers aiding in HCC diagnosis, treatment and prognostic assessment of HCC. It also explores the role of DNA methylation in regulating HCC's malignant progression and sorafenib resistance, alongside elaborating the therapeutic effects of DNA methyltransferase inhibitors on HCC. A detailed examination of these aspects underscores the significant research on DNA methylation in tumour cells to elucidate malignant progression mechanisms, identify diagnostic markers and develop new tumour‐specific inhibitors for HCC.

**Key points:**

- A comprehensive summary of various aspects of DNA methylation, such as its mechanism, detection methods and biomarkers aiding in diagnosis and treatment.
- The role of DNA methylation in regulating hepatocellular carcinoma's (HCC) malignant progression and sorafenib resistance, alongside elaborating therapeutic effects of DNA methyltransferase inhibitors.
- Deep research on DNA methylation is critical for discovering novel tumour‐specific inhibitors for HCC.

## Graphical abstract

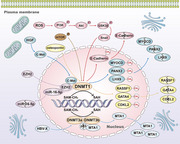

## 1 BACKGROUND

Hepatocellular carcinoma (HCC) is the predominant form of primary liver cancer, ranking second in global mortality rates and posing significant health threats.^1^, ^2^, ^3^ An approximate death rate of more than 0.38 million individuals is projected annually in China due to liver cancer.^4^ The aetiology of HCC mainly includes factors such as alcohol consumption, smoking, hepatitis B and C infections, diabetes and aflatoxin exposure.^5^ Current treatment regimens for HCC encompass surgical intervention, sorafenib‐based chemotherapy and interventional therapy, among others. However, the prognosis for HCC remains dismal, with recurrence and metastasis as leading causes of mortality.^6^ Consequently, it is imperative to identify effective diagnostic biomarkers and specific therapeutic targets for HCC while developing novel HCC inhibitors.

Numerous studies investigating the diagnosis, treatment and prognosis of HCC have underscored the significant association of DNA methylation with HCC.^7^, ^8^ For instance, the methylation rate of glutathione S‐transferase P1 (*GSTP1*) in HCC is substantially higher, compared to tissues of nodular dysplasia and liver cirrhosis, correlating strongly with poor patient prognosis and suggesting its potential as a diagnosis biomarker for HCC.^9^ Moreover, DNA methylation diminishes the mRNA and protein expression of caspase‐8 and E‐cadherin (Cadherin 1, *CDH1*) genes, enhancing the anti‐apoptotic capability of tumour cells and promoting invasion and metastasis, respectively.^10^ Furthermore, promising anti‐tumour benefits have been demonstrated with small molecule inhibitors of DNA methyltransferase.^11^ Given that DNA methylation promotes the malignant progression of HCC, it emerges as a potential target for clinical treatment and lays the groundwork for developing novel inhibitors. This review aims to comprehensively summarise various aspects of DNA methylation, including its mechanism, detection methods and biomarkers aiding in the diagnosis, treatment and prognostic assessment of HCC. Furthermore, it explores the role of DNA methylation in regulating the occurrence and development of HCC while elaborating the therapeutic effects of DNA methyltransferase inhibitors (DNMTi). These findings are anticipated to stimulate new ideas and advance clinical therapies for HCC.

## 2 MECHANISM OF DNA METHYLATION

Previous research has highlighted the significant role of epigenetic modifications in the occurrence and development of solid tumours. Typically, these alterations manifest in three ways: (1) DNA hypermethylation, which occurs in promoter islands inhibits gene expression; DNA hypermethylation, which occurs in gene body islands may activate the expression of some genes (DNA hypermethylation mentioned in this review almost all occurs in promoter islands and inhibits gene expression). (2) DNA hypomethylation, which increases genomic instability; and (3) histone modification, which influences the spatial conformation of chromosomes.^12^ Among these, DNA hypermethylation inactivates tumour suppressor gene expression. Meanwhile, DNA hypomethylation of the entire genome is generally recognised as an early pathological alteration in the tumour growth process.^13^

**FIGURE 1:**
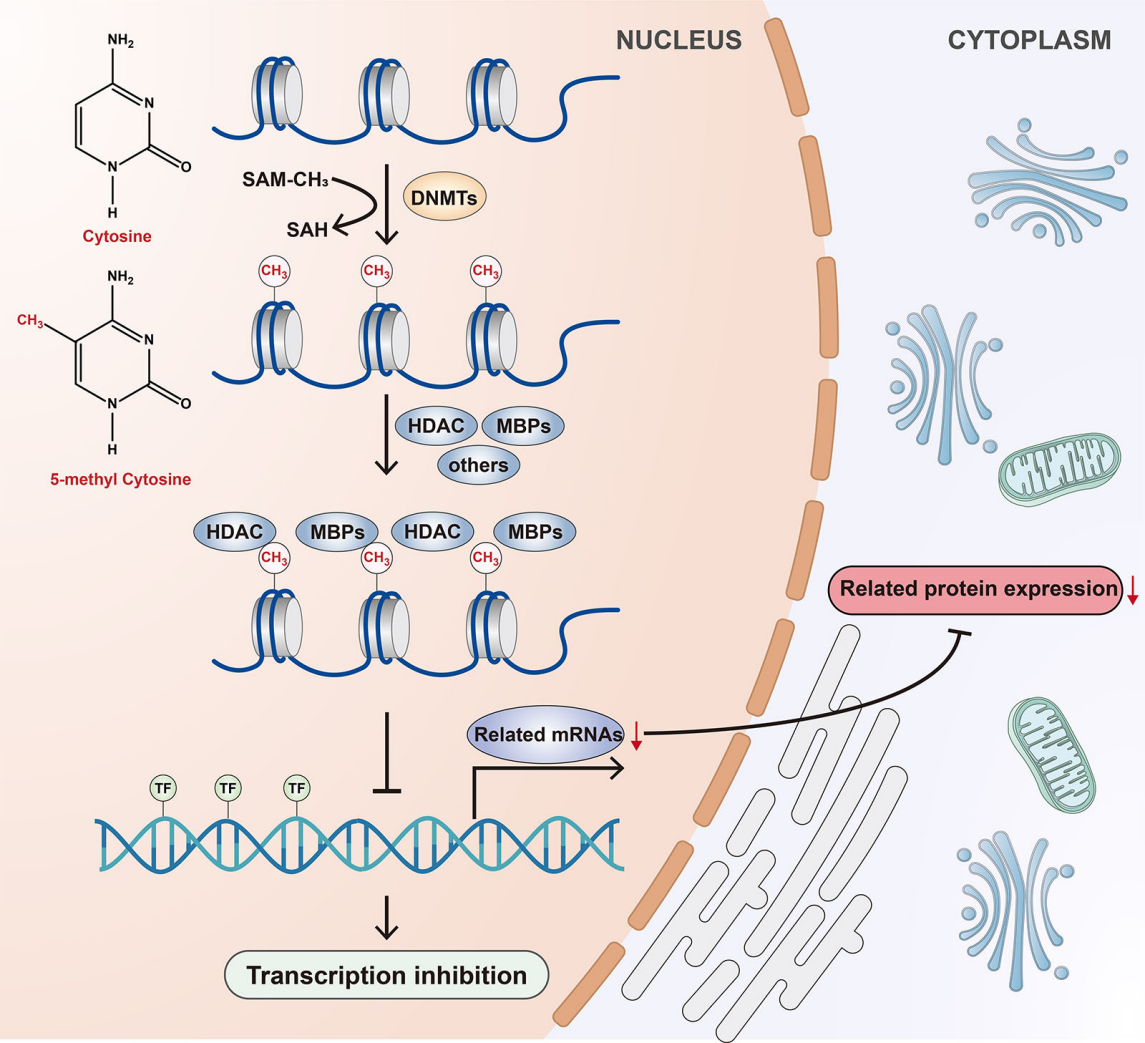
Process of DNA methylation and its inhibitory effect on gene transcription. To achieve the DNA methylation, DNA methyltransferase (DNMT) first catalyses the addition of a methyl group, donated by S‐adenosyl methionine (SAM‐CH3), to the fifth carbon of cytosine, resulting in the conversation of methylated SAM‐CH3 to non‐methylated S‐adenosylhomocysteine (SAH) and the formation of stable 5‐methyl cytosine structures. Then, after a methyl group occupies a cytosine position on DNA, the methylated cytosine‐phosphate‐guanine (CpG) attracts inhibitory proteins (such as histone deacetylase [HDAC], methyl binding proteins [MBPs] and others) that impede the binding of transcription factors to DNA promoters, thereby suppressing gene transcription and related protein expression.

In DNA methylation, the enzyme DNA methyltransferase catalyses the addition of a methyl group, donated by S‐adenosyl methionine (SAM‐CH3), to the fifth carbon of cytosine‐phosphate‐guanine (CpG) dinucleotide, resulting in the formation of stable 5‐methylcytosine (5mC) structures. This formation constitutes the sole covalent alteration to DNA in mammals.^14^ When a methyl group occupies a cytosine position on DNA, the methylated CpG attracts inhibitory proteins (such as histone deacetylase [HDAC], methyl binding proteins and others) that impede the binding of transcription factors (such as E2F transcription factor 1 and nuclear factor‐kappa B [NF‐κB]) to gene promoters, thereby suppressing gene transcription.^15^ Above process is also presented in Figure 1. CpG dinucleotides are unevenly distributed across the human genome, with approximately one CpG dinucleotide for every 80 dinucleotides. Moreover, the CpG‐rich regions, known as CpG islands, constitute approximately 1%–2% of the whole genome.^16^ These islands are present in approximately 45 000 promoter regions and transcription start sites of various genes. Moreover, about half of all genes contain CpG islands in their 5′ region, encompassing promoter regions and transcription start sites.^17^ This structural organisation reflects the close relationship between DNA promoter methylation and gene transcription. When transcription factors exert an inhibitory role, DNA methylation occupies the binding site of these transcription factors and may even enhance the transcription of related genes, such as some oncogenes.^18^ Beyond its influence on gene transcription, DNA methylation is crucial for maintaining normal embryonic development. Animal models have demonstrated that DNA methyltransferase 1 (DNMT1), DNMT3a and DNMT3b are essential for the normal embryonic development of mice. Furthermore, deficiencies in DNMT1 and DNMT3b are embryonically lethal for mice, while DNMT3a‐deficient mice survive the embryonic stage and succumb shortly after that.^19^ Since the sole focus is on the active DNA methyltransferases in humans, DNA methyltransferase 3c and DNA methyltransferase 3l are not discussed. This review highlights the necessity of maintaining methylation homeostasis in mammals. Moreover, in tumour cells, DNA is hypomethylated, indicating a reduced level of DNA methylation, compared to normal cells. This decline in DNA methylation levels leads to the upregulation of oncogene transcription, a decrease in nuclear stability and the development of tumours.^20^

Researchers have long grappled with the transmission of epigenetic information during cell division. Extensive investigations into DNA methylation have revealed that this modification may undergo complete duplication during the S phase of mitosis. The concept of ‘half‐reserved replication’ mirrors DNA replication, ensuring that the methylation pattern is faithfully transmitted to progeny cells. Consequently, DNA methylation consistently contributes to silencing specific essential genes, regardless of cell division.^21^ While DNA methylation exhibits distinct patterns during early embryonic development and germ cell maturation, it remains relatively stable in adult animals, undergoing minimal modifications.^22^ It is imperative to emphasise that aberrant DNA methylation alterations can lead to irreversible epigenetic disorders, transforming normal cells into tumour cells and culminating in the development of malignancies. Moreover, this process does not alter genetic information; it merely disrupts epigenetic modification. This observation indicates the role of DNA methylation‐mediated epigenetic changes as the earliest aetiological abnormalities in tumour formation.

Meanwhile, owing to HCC as one of the most heterogeneous cancers because of its various etiologies,^23^, ^24^ there are specific methylation patterns according to the cause. First, Hlady et al. reported the aberrant DNA methylation patterns under the heterogeneity of precancerous lesions of HCC.^25^ They detected the genome‐wide DNA methylation and copy number variation via the Infinium 450K in the regenerative nodules from the livers of single patients. Within these regenerative nodules, some of them with a higher frequency of DNA methylation changes have significantly lower variations of copy number, while others do not. These nodules were also evaluated with higher scores based on epigenetic changes and related to increased cell proliferation ability. Furthermore, these epigenetically aggressive nodules were enriched for oncogenes involved in the development of HCC. All these results indicated that there were different methylation patterns under the genetic heterogeneity, and different nodules had different enrichment of epigenetic and genetic components, which would contribute to different HCC progression. In addition, histone H4 methylation (H4 M) modification is also a key epigenetic modification and is involved in the development of HCC.^26^, ^27^ It also has different patterns under the heterogeneity of HCC. Yu et al. enrolled 2305 HCC samples and performed consensus clustering analysis, then they identified three distinct H4 M modification patterns.^28^ Furthermore, they also created H4Mscore via a principal component analysis algorithm to quantify the different H4 M modification patterns of every individual tumour that predicted and reflected the heterogeneity of HCC. Specifically, patients with a low H4Mscore had a better response to immunotherapy and a better survival outcome. Therefore, evaluating and identifying the H4 M modification patterns of individual HCC tumours may contribute to study the tumour heterogeneity and novel therapeutic strategies. Besides, by collecting 738 HCC samples, Meunier et al. developed a framework called methylation signature analysis with independent component analysis, which totally identified 13 stable methylation patterns.^29^ They included patterns related to sex, age, specific driver events and molecular subgroups for HCC. This study clearly elucidated the diversity of HCC methylomes and also indicated the strong tumour heterogeneity of HCC.

## 3 DETECTION METHODS OF DNA METHYLATION

Effective detection methods for DNA methylation are crucial for tumour diagnosis. Polymerase chain reaction (PCR) has been the most commonly used method; however, various diagnostic approaches are available for different forms of methylation analysis.

### 3.1 Global methylation analysis

Methods for global methylation analysis encompass radiolabelled methyl groups, methylation‐sensitive restriction endonuclease (MSRE), enrichment of methyl‐cytosine‐binding proteins, PCR, DNA methylation detection through repeat sequences, liquid chromatography‐mass spectrometry and biotin‐streptavidin‐horseradish peroxidase‐mediated colorimetry assays. Recent advancements have also introduced widely available techniques such as the HumanMethylation450k BeadChip assay,^30^, ^31^, ^32^ the MethylationEPIC BeadChip assay,^33^, ^34^, ^35^ No End‐repair Enzymatic Methyl‐seq (NEEM‐seq) and genome‐wide single‐cell methylation assays.^36^, ^37^, ^38^, ^39^, ^40^

### 3.2 Genome‐scale methylation analysis

Various methods are employed for genome‐scale methylation analysis, including methylation‐specific PCR (MSP), direct MSP, nested MSP, bisulfite genomic sequencing, methylight,^41^ combined bisulphate restriction analysis (COBRA), methylated DNA‐immunoprecipitation sequencing (MeDIP‐Seq), methylated DNA immunoprecipitation‐chip, methylation‐sensitive restriction enzymes‐based quantitative PCR, MSRE‐based Southern blot, sulfite treatment and MassArray, gel electrophoresis^42^ and Infinium HumanMethylation450 BeadChip.

### 3.3 DNA methylation kinetics analysis

Methods such as methylation‐assisted bisulfite sequencing (MAB‐Seq), endonuclease AbaSI sequencing and reduced‐representation MAB‐seq are currently available for this type of analysis.^43^, ^44^, ^45^ Traditional MSP is susceptible to DNA methylation detection among these. By utilising specific primers, MSP selectively amplifies methylation alleles, enabling the detection of low DNA methylation levels.^46^ Moreover, quantitative detection methods based on MSP, such as single‐molecule real‐time (SMRT) sequencing and MethyLight, offer higher sensitivity, specificity and powerful capabilities for detecting DNA methylation. MeDIP‐Seq and BSP are two concurrent feasible methods for detecting DNA methylation. While MeDIP‐Seq utilises anti‐5mC antibodies to generate 10^7^ reads, BSP has emerged as a gold standard for methylation sequencing, capable of generating 10^8^ methylated cytosines with single nucleotide resolution.^47^, ^48^ Although various approaches exist for detecting DNA methylation, each has distinct applications and associated costs. However, the absence of standardised and reproducible detection technologies compromises DNA detection techniques.^49^ Therefore, extensive standardisation of the DNA methylation detection method is essential to ensure a guaranteed and effective screening and the identification of tumour biomarkers.

## 4 DNA METHYLATION BIOMARKERS FOR DIAGNOSIS, TREATMENT AND PROGNOSTIC ASSESSMENT OF PATIENTS WITH HCC

DNA methylation biomarkers aid in diagnosing, treating and prognostic evaluating secondary HCC and are primarily categorised into tissue and blood or bodily fluid biomarkers. Both types of biomarkers are elaborated in detail below.

### 4.1 DNA methylation biomarkers in tissues

#### 4.1.1 Hypermethylation events

Investigations discussing the biomarkers in HCC tissues have revealed a strong association of hypermethylation of the deuterosome assembly protein 1 (*DEUP1*) gene promoter with lymph node metastatic status and tumour differentiation in patients with HCC.^50^ Moreover, DNA hypermethylation‐mediated inhibition of keratin 19 expression is crucially related to poor survival outcomes of patients with HCC.^51^ The expression of chaperonin containing T‐complex 1 subunit 3 (CCT3) protein is negatively correlated with DNA methylation levels. Pathway enrichment analysis reveals that CCT3 might play a crucial role in HCC tumour staging and patient prognosis by regulating the cell cycle and the DNA replication pathway.^52^ Formiminotransferase cyclodeaminase (FTCD) was also regulated by DNA promoter hypermethylation. Low FTCD expression is closely related to poor prognosis, high alpha‐fetoprotein (AFP) level and deep degree of vascular invasion in patients with HCC.^53^ In HCC tissues, the *GSTP1* methylation ratio exceeds that in nodular dysplasia and liver cirrhosis tissues, strongly correlating with poor prognosis.^9^ Hypermethylation of the *CDH1* gene promoter, indicative of epithelial‐mesenchymal transition, is related to the malignant progression of HCC.^54^ Tissue hypermethylation in the promoter of the long non‐coding RNA (LncRNA) PDZD7 gene activates the stem cell characteristics of HCC and renders it less susceptible to anticancer drugs, making it a potential clinical biomarker and therapeutic target.^55^ In HCC tissues, hypermethylation of the tyrosine kinase (*FES*) promoter is closely associated with tumour size, AFP level and tumour differentiation degree, suggesting its potential as a prognostic biomarker.^56^ Similarly, hypermethylation of the argininosuccinate synthase 1 *(ASS1)* promoter in HCC tissue correlates closely with the invasion and migration of HCC, indicating its potential for diagnosis and treatment.^57^ Moreover, hypermethylation of the *CXCL2* promoter in HCC tissue is linked to tumour immunity, making it a potential target for HCC diagnosis and treatment.^58^ The hypermethylation event at the vasoactive intestinal peptide receptor 1 (*VIPR1*) promoter in HCC tissues is significantly related to poor prognoses in patients with HCC.^59^ Similarly, hypermethylation of the zinc‐finger protein 382 (*ZNF382*) promoter in HCC tissue may contribute to malignant progression, typically linked with a worse prognosis.^60^ Zhou et al. discovered that hypermethylation of *CDKN2A* gene in liver cancer tissue is frequently associated with hepatitis B and C viral infections, liver cirrhosis status and age, suggesting its potential as a diagnostic and therapeutic target for HCC.^61^ Chen et al. found that hypermethylation of the alpha‐1A adrenergic receptor (*ADRA1A*) gene promoter was significantly correlated with alcohol consumption and AFP level, indicating its diagnostic value for HCC. The receiver operating characteristic (ROC) curve demonstrated that *ADRA1A* could effectively distinguish HCC tissues from adjacent normal tissues.^62^ Zhao et al. observed that telomerase reverse transcriptase (*TERT*) gene promoter hypermethylation in HCC was associated with AFP level, tumour size, intrahepatic metastasis and TNM stage of patients with HCC. Simultaneously, hypermethylation of this gene is typically associated with a worse survival outcome in patients with HCC.^63^ Furthermore, some studies suggest that DNA hypermethylation can upregulate the expression of the *TP73* gene in HCC, indicating its potential as a biomarker for HCC diagnosis.^64^ The hypermethylation level of the adenomatous polyposis coli (*APC*) gene in HCC is higher in HCC patients than in healthy donors. Furthermore, the DNA methylation level of the *APC* gene is also higher in those HCC patients with TNM III and IV stages, compared to those with TNM I and II stages, and the methylation status of the *APC* gene is significantly associated with lymph node metastasis and tumour size, suggesting its potential as a prognostic biomarker for HCC.^65^ Fu et al. found aberrant hypermethylation changes in Homeobox A1 (*HOXA1*), *CLEC11A*, AK055957 and TSPY‐like 5 (*TSPYL5*) in non‐cirrhotic HCC tissues, compared to other benign lesions, suggesting that they could serve as early detectable biomarkers for non‐cirrhotic HCC.^66^ Furthermore, other hypermethylation biomarkers may also aid in the clinical diagnosis of HCC, including hypermethylated paired box 6 (*PAX6*),^67^ *PAX5*, *SPDYA*, proenkephalin (*PENK*), kelch‐like 35 (*KLHL35*), *TSPYL5*,^68^ *ACP1* and *BMP4*.^69^ Table 1 summarises the main DNA hypermethylation biomarkers in tissues that aid HCC diagnosis, treatment and prognosis prediction.

**TABLE 1:** DNA hypermethylation biomarkers in tissues to aid hepatocellular carcinoma (HCC) diagnosis, treatment and prognosis prediction.

No.	Gene name	Expression	Application	Reference
1	*DEUP1*	Decreased	Biomarker to aid HCC diagnosis	^50^
2	*Keratin 19*	Decreased	Linked to poor prognosis of HCC patients	^51^
3	*CCT3*	Decreased	Involved in HCC tumour staging and patient's prognosis by regulating the cell cycle and the DNA replication pathway	^52^
4	*FTCD*	Decreased	Used as a diagnostic biomarker and a potential therapeutic target in HCC. Low expression of FTCD is closely related to the poor prognosis, alpha‐fetoprotein (AFP) level, larger tumour size and vascular invasion of HCC	^53^
5	*GSTP1*	Decreased	Linked with poor prognosis of patients with HCC	^9^
6	*CDH1*	Decreased	Related to the malignant progression of HCC	^54^
7	*Lnc‐PDZD7*	Decreased	Used as a clinical marker and potential therapeutic target	^55^
8	*FES*	Decreased	Related to tumour size, AFP level and tumour differentiation degree and may serve as a prognostic biomarker	^56^
9	*ASS1*	Decreased	Related to the invasion and migration of HCC and used for the diagnosis and treatment of HCC	^57^
10	*CXCL2*	Decreased	Related to tumour immunity making it a possible target for diagnosis and treatment of HCC	^58^
11	*VIPR1*	Decreased	Linked to poor prognosis in HCC patients	^59^
12	*ZNF382*	Decreased	Linked to a poor prognosis of patients with HCC	^60^
13	*CDKN2A*	Decreased	Linked to hepatitis B and C virus infections, liver cirrhosis status, age and may serve as a potential target for diagnosis and treatment of HCC	^61^
14	*ADRA1A*	Decreased	Related to alcohol consumption and AFP level, indicating its high diagnostic value for HCC	^62^
15	*TERT*	Decreased	Related to AFP level, tumour size, intrahepatic metastasis, TMN stage and poor prognosis of patients with HCC	^63^
16	*TP73*	Decreased	Used as a biomarker for the diagnosis of HCC	^64^
17	*APC*	Decreased	Used as a prognostic biomarker for patients with HCC	^65^
18	*HOXA1*	Decreased	Serve as potentially early detectable biomarkers for non‐cirrhotic HCC	^66^
19	*CLEC11A*	Decreased	Serve as potentially early detectable biomarkers for non‐cirrhotic HCC	^66^
20	*TSPYL5*	Decreased	Serve as potentially early detectable biomarkers for non‐cirrhotic HCC	^66^
21	*PAX6*	Decreased	Assist in the clinical diagnosis of HCC	^67^
22	*PAX5*	Decreased	Assist in the clinical diagnosis of HCC	^68^
23	*SPDYA*	Decreased	Assist in the clinical diagnosis of HCC	^68^
24	*PENK*	Decreased	Assist in the clinical diagnosis of HCC	^68^
25	*KLHL35*	Decreased	Assist in the clinical diagnosis of HCC	^68^
26	*TSPYL5*	Decreased	Used as potentially detectable biomarkers in HCC tissues	^68^, ^69^
27	*ACP1*	Decreased	Used as potentially detectable biomarkers in HCC tissues	^69^
28	*BMP4*	Decreased	Used as potentially detectable biomarkers in HCC tissues	^69^

Abbreviations: ACP1, acid phosphatase locus 1; *ADRA1A*, alpha‐1A adrenergic receptor; *APC*, adenomatous polyposis coli; *ASS1*, argininosuccinate synthase 1; BMP4, Bone Morphogenetic Protein 4; *CCT3*, chaperonin containing T‐complex 1 subunit 3; *CDH1*, E‐cadherin; *CDKN2A*, cyclin‐dependent kinase inhibitor 2A; *CLEC11A*, C‐Type Lectin Domain Containing 11A; *CXCL2*, C‐X‐C motif chemokine ligand 2; *DEUP1*, deuterosome assembly protein 1; *FES*, tyrosine kinase; *FTCD*, formiminotransferase cyclodeaminase; *GSTP1*, glutathione S‐transferase P1; *HCCS1*, Hepatocellular carcinoma suppressor 1; *HOXA1*, homeobox A1; IGF‐II, insulin‐like growth factor‐II; *KLHL35*, kelch‐like 35; *LINE‐1*: Long interspersed nuclear element‐1; *NLRP2* NLR Family Pyrin Domain Containing 2; *NLRP3*, NLR Family Pyrin Domain Containing 3; *NLRP7*, NLR Family Pyrin Domain Containing 7; *PAX5*, paired box 5; *PAX6*, paired box 6; *PENK*, proenkephalin; *RASSF1A*, ras‐association domain family 1A; SOCS‐1, suppressor of cytokine signaling 1; *SPDYA*, Speedy A; *TERT*, telomerase reverse transcriptase; *TP73*, Tumor Protein P73; *TSPYL5*, TSPY Like 5; *VIPR1*, vasoactive intestinal peptide receptor 1; *ZNF382*, zinc‐finger protein 382.

#### 4.1.2 Hypomethylation events

Hypomethylation of the growth arrest‐specific 5 (GAS5) promoter in HCC tissues leads to the high expression of LncRNA GAS5, serving as an independent predictor of recurrence‐free survival.^70^ Promoter hypomethylation of tonsoku‐like DNA repair protein in HCC tissue regulates the cell cycle, suggesting its potential as a prognostic biomarker for patients with HCC.^71^ Hypomethylation of the CCAAT/enhancer‐binding protein‐beta (*C/EBP‐β*) promoter is associated with the activation of oncogenes during early tumour formation and poor prognosis of patients.^72^ HCC tissue with hypomethylated glycerol‐3‐phosphate dehydrogenase 1‐like (*GPD1L*) promoters exhibits microvascular invasion and poor patient prognosis.^73^ Hypomethylation of the aurora kinase A (*AURKA*) and Activator protein‐1 (AP‐1) transcription factor subunit (*FOS*) promoters in HCC tissue is linked to inflammation. It may serve as a therapeutic target for hepatitis C virus (HCV)‐positive patients with HCC.^74^ Hypomethylation of the zinc‐finger CCHC‐type containing 13 (*ZCCHC13*) promoters in HCC tissue may be a driving oncogene in tumour formation.^75^ Hypomethylation of the inositol 1,4,5‐trisphosphate receptor (*ITPR3*) promoter significantly contributes to HCC development.^76^ Promoter hypomethylation of tubulin, beta 2B class IIB (*Tubb2b*) in HCC tissue promotes HCC progression and has diagnostic value.^77^ Hypomethylation of the fork‐head box K1 (*FOXK1*) promoter in HCC tissue regulates tumour metastasis and could be a prognostic biomarker for patients with HCC.^78^ Hypomethylation of the cell division cycle‐associated 5 (*CDCA5*) promoter in HCC tissue can promote cell proliferation and holds potential as a target for diagnosing, treating and prognosticating HCC.^79^ Similarly, the hypomethylation of *LINE‐1* in HCC tissues promotes the transition from normal liver cells to tumour cells and serves as a prognostic biomarker for patients with HCC.^80^ Ribosomal Protein S2 (*RPS2*) promoter hypomethylation potentially offers prognostic insights for individuals with HCC.^81^ Li et al. observed that DNA hypomethylation‐mediated upregulation of coiled‐coil domain‐containing protein 50 (*CCDC50*) was significantly associated with adverse survival outcomes in patients with HCC, suggesting its potential as an independent prognostic biomarker.^82^ Furthermore, Fang et al. suggested that DNA hypomethylation‐mediated elevated expression of lymphoid‐specific helicase (*HELLS*) might be a potential biomarker for prognostically assessing HCC.^83^ Zhang et al. identified hypomethylation in the gene bodies of pyroptosis‐related genes, *NLRP7*, *NLRP2* and *NLRP3* as promising biomarkers for early detection, recurrence monitoring and prognostic assessment of HCC.^84^ Table 2 summarises the main DNA hypomethylation biomarkers in tissues that aid HCC diagnosis, treatment and prognosis prediction.

**TABLE 2:** DNA hypomethylation biomarkers in tissues to aid HCC diagnosis, treatment and prognosis prediction.

No.	Gene name	Expression	Application	Reference
1	*GAS5*	Increased	An independent predictor of recurrence‐free survival for patients with HCC	^70^
2	*TONSL*	Increased	Employed as a prognostic biomarker for patients with HCC	^71^
3	*C/EBP‐β*	Increased	Linked with the activation of oncogenes during early tumour formation and related to poor prognosis of patients with HCC	^72^
4	*GPD1L*	Increased	Characterised by microvascular invasion and poor patient outcomes	^73^
5	*AURKA*	Increased	Related to inflammation and can be used as a therapeutic target for HCV‐positive HCC patients	^74^
6	*FOS*	Increased	Related to inflammation and can be used as a therapeutic target for HCV‐positive HCC patients	^74^
7	*ZCCHC13*	Increased	Used as a driving oncogene in the process of HCC formation	^75^
8	*ITPR3*	Increased	One of the major factors contributing to the development of HCC	^76^
9	*Tubb2b*	Increased	Promote the progress of HCC and has a high diagnostic value	^77^
10	*FOXK1*	Increased	Serve as a prognostic biomarker for patients with HCC	^78^
11	*CDCA5*	Increased	Used as a target for diagnosis, treatment and prognosis prediction of HCC	^79^
12	*LINE‐1*	Increased	Promote the transformation of normal liver cells into tumour cells and can be used as a biomarker of the prognosis for HCC patients	^80^
13	*RPS2*	Increased	Serve as a prognostic biomarker for patients with HCC	^81^
14	*CCDC50*	Increased	Serve as an independent prognostic biomarker for patients with HCC	^82^
15	*HELLS*	Increased	Serve as a potential biomarker for prognostic prediction of HCC	^83^
16	*NLRP7*	Increased	A promising biomarker for the early detection, monitoring recurrence and prognosis prediction of HCC	^84^
17	*NLRP2*	Increased	A promising biomarker for the early detection, monitoring recurrence and prognosis prediction of HCC	^84^
18	*NLRP3*	Increased	A promising biomarker for the early detection, monitoring recurrence and prognosis prediction of HCC	^84^

Abbreviations: *AURKA*, aurora kinase A; *C/EBP‐β*, CCAAT/enhancer‐binding protein‐beta; *CCDC50*, coiled‐coil domain‐containing protein 50; *CDCA5*, cell division cycle‐associated 5; *FOS*, AP‐1 transcription factor subunit; *GAS5*, growth arrest‐specific 5; *GPD1L*, glycerol‐3‐phosphate dehydrogenase 1‐like; *HELLS*, lymphoid‐specific helicase; *ITPR3*, inositol 1,4,5‐trisphosphate receptor; *TONSL*, Ribosomal Protein S2; *Tubb2b*, tubulin, beta 2B class IIB; *FOXK1*, fork‐head box K1; *ZCCHC13*, zinc‐finger CCHC‐type containing 13.

### 4.2 DNA methylation biomarkers in blood or body fluids

DNA methylation biomarkers in blood or body fluids are also crucial in predicting and diagnosing HCC. Although AFP alone is commonly used, its sensitivity and specificity are suboptimal.^85^, ^86^, ^87^ Consequently, combining AFP with other biomarkers such as des‐gamma‐carboxy prothrombin,^88^ glypican‐3^89^ and squamous cell carcinoma antigen has been explored to enhance sensitivity and specificity.^90^, ^91^

Similarly, studies have explored the combination of DNA methylation‐related biomarkers with AFP for HCC prediction and diagnosis. The hypermethylation level of the liver tumour suppressor gene G‐protein‐coupled bile acid receptor (*Gpbar1*/*TGR5*) promoter in the serum of patients with HCC is substantially elevated, compared to patients with chronic hepatitis B and healthy controls. This elevated level exhibits high sensitivity in diagnosing HCC and demonstrates increased diagnostic accuracy when combined with AFP levels in the blood for HCC diagnosis.^92^ In the serum of patients with HCC, hypermethylation of organic cation transporter 1 (*OCT1*, also known as *SLC22A1*) and Kruppel‐like factor 4‐003 (*KLF4‐003*) promoters leads to reduced expression of SLC22A1 and KLF4‐003 proteins, making them potential biomarkers for HCC diagnosis.^93^, ^94^ Another study identified hypermethylation of the natural killer cell receptor group 2D (*NKG2D*) gene promoters in peripheral blood mononuclear cells (PBMCs) of patients with HCC, suggesting its utility as a non‐invasive biomarker for detecting HCC.^95^ Similarly, hypermethylation of the glutathione S‐transferases (*GST1*) promoter in serum is associated with an increased risk of adverse prognosis in patients with HCC.^96^ A combination analysis of hypermethylated *RASSF1A* or hypermethylated*SOCS‐1* in peripheral blood with AFP in serum can also be applied as a non‐invasive biomarker for detecting HCC.^97^, ^98^ Abou Zeid et al. also found *RASSF1A* gene promoter hypermethylation in plasma can serve as a biomarker for chronic viral hepatitis C‐related HCC.^99^ Hypermethylation of the *HCCS1* gene promoter in patients’ serum shows promise as a biomarker for the diagnosis and prognosis of patients with HCC.^100^ Ji et al. found that hypermethylation of the metallothionein 1M (*MT1M*) and metallothionein 1G (*MT1G*) gene promoters in serum can serve as diagnostic biomarkers for HCC and are associated with vascular invasion and metastasis of HCC.^101^ Hypomethylation of interleukin‐17 (*IL‐17*) and oncogenic *IGF‐II* promoters in patients’ serum can be utilised as biological biomarkers for diagnosing and treating HCC.^102^, ^103^ Moreover, Xie et al. demonstrated that hypermethylation of the SH3 domain GRB2‐like endophilin interacting protein 1 (*SGIP1*) gene in plasma holds higher diagnostic value for HCC and is typically associated with worse clinical outcomes in HCC patients.^104^ Qian et al. discovered significantly elevated cyclin D2 (*CCND2*) gene promoter hypomethylation levels in plasma and peripheral blood mononuclear cells of HCC patients, compared to those of healthy controls. When combined with AFP, the analysis of the *CCDN2* gene demonstrated increased sensitivity and area under the curve for the diagnosis of HCC, compared to AFP alone, indicating the potential of *CCDN2* gene promoter methylation in plasma to predict the progression of HCC.^105^ Sun et al. observed promoter hypermethylation of signal transduction adaptor protein 1 (*STAP1*) in peripheral blood T cells, suggesting its potential as a prognostic biomarker for HCC patients with tumour sizes less than 5 cm.^106^ In PBMCs, the promoter methylation level of F‐box protein 43 (*FBXO43*) was lower in patients with HCC than in healthy controls. The ROC analysis revealed its superior ability to differentiate HCC from hepatitis, even outperforming serum AFP levels.^107^ Saeki et al. highlighted methylated‐septin‐9 (*m‐SEPT9*) in serum as a significant predictor of poor survival outcomes in patients with HCC treated with molecularly targeted agents such as sorafenib or lenvatinib.^108^ Bai et al. utilised 850K methylation arrays to develop a blood‐based multi‐marker HCC detection panel (HepaClear), showing high sensitivity in screening and diagnosing HCC in at‐risk populations.^109^ Udali et al. found that DNA promoter methylation regulates hepcidin antimicrobial peptide (*HAMP*) gene expression. Hypermethylation of the *HAMP* gene promoter in HCC inhibits gene expression, affecting the concentration of hepcidin and iron ions in the blood of patients with HCC.^110^ Moreover, clinical trials validated several plasma methylated DNA markers for early HCC detection (empty spiracles homeobox 1 [*EMX1*], *HOXA1*, AK055957, *CLEC11A*, endothelin‐converting enzyme 1 [*ECE1*] and phosphofructokinase [*PFK*]).^111^ Mezzacappa et al. explored methylation markers of HCC in salivary DNA, revealing differentially methylated genes encoding tumour suppressors (*PRDM2*, *RASSF1/5 RUNX3* and *p15/16*,), cell cycle regulators (*TP73* and *DAPK1*) and DNA repair regulators (O^6^‐methylguanine DNA methyltransferase [*MGMT*] and *GSTP1*). Salivary DNA was proposed as a potential alternative to blood samples for novel DNA‐based HCC diagnostic screening tests.^112^ Table 3 summarises the main biomarkers of DNA methylation in blood or body fluids that can aid HCC diagnosis, treatment and prognosis prediction.

**TABLE 3:** DNA methylation biomarkers in blood or body fluids to aid HCC diagnosis, treatment and prognosis prediction.

No.	Gene name	DNA methylation status	Expression	Specimen	Application	Reference
1	*TGR5*	Hypermethylation	Decreased	Serum	Serve as a diagnostic biomarker for patients with HCC	^92^
2	*SLC22A1*	Hypermethylation	Decreased	Serum	Used as a biomarker for HCC diagnosis	^93^
3	*KLF4‐003*	Hypermethylation	Decreased	Serum	Used as a biomarker for HCC diagnosis	^94^
4	*NKG2D*	Hypermethylation	Decreased	PBMCs	Used as a non‐invasive biomarker to detect HCC	^95^
5	*GST1*	Hypermethylation	Decreased	Serum	Associated with an increased risk of poor prognosis in HCC patients	^96^
6	*RASSF1A*	Hypermethylation	Decreased	Peripheral blood	Combined with AFP in serum can be applied as a non‐invasive biomarker for detecting HCC	^97^, ^98^
7	*RASSF1A*	Hypermethylation	Decreased	Plasma	Serve as a biomarker for chronic viral hepatitis C‐related HCC	^99^
8	*SOCS‐1*	Hypermethylation	Decreased	Peripheral blood	Combined with AFP in serum can be applied as a non‐invasive biomarker for detecting HCC	^97^
9	*HCCS1*	Hypermethylation	Decreased	Serum	Utilised as a biomarker for the diagnosis and prognosis of HCC patients	^100^
10	*MT1M*	Hypermethylation	Decreased	Serum	Used as a diagnostic biomarker of HCC and closely related to vascular invasion and metastasis of HCC	^101^
11	*MT1G*	Hypermethylation	Decreased	Serum	Used as a diagnostic biomarker of HCC and closely related to vascular invasion and metastasis of HCC	^101^
12	*IL‐17*	Hypomethylation	Increased	Serum	Utilised as biological biomarkers to diagnose and treat HCC	^102^
13	*IGF‐II*	Hypomethylation	Increased	Serum	Utilised as biological biomarkers to diagnose and treat HCC	^103^
14	*SGIP1*	Hypermethylation	Decreased	Plasma	Possess a higher diagnosis value for HCC and typically linked with a poor prognosis of patients with HCC	^104^
15	*CCND2*	Hypomethylation	Increased	Plasma and PBMCs	Combined analysis of the methylation status of CCDN2 and AFP possessing a higher sensitivity and area under the curve for the diagnosis of HCC	^105^
16	*STAP1*	Hypermethylation	Decreased	Peripheral blood T cells	Serve as a potential prognostic marker for patients with HCC whose tumour size was less than 5 cm	^106^
17	*FBXO43*	Hypomethylation	Increased	PBMCs	Possess an excellent differentiating ability of HCC from chronic hepatitis.	^107^
18	*SEPT9*	Hypermethylation	Decreased	Serum	Could be a significant predictor of poor overall survival in patients with HCC treated with molecular‐targeted agents like sorafenib or lenvatinib	^108^
19	HepaClear^a^	–	–	Blood	Present high sensitivity for the screening and diagnosis of HCC from an at‐risk population	^109^

Abbreviations: *CCND2*, cyclin D2; *FBXO43*, F‐box protein 43; *GST1*, glutathione S‐transferases; *IL‐17*, interleukin‐17; *KLF4‐003*, Kruppel‐like factor 4‐003; *MT1G*, metallothionein 1G; *MT1M*, metallothionein 1M; *NKG2D*, natural killer cell receptor group 2D; *SEPT9*, septin‐9; *SLC22A1*, organic cation transporter 1; *SGIP1*, SH3 domain GRB2‐like endophilin interacting protein 1; *STAP1*, signal transduction adaptor protein 1; *TGR5*, G‐protein‐coupled bile acid receptor.

^a^ A blood‐based multi‐marker HCC detection panel (HepaClear).

Moreover, methylated circulating tumour DNA (ctDNA) and cell‐free DNA (cfDNA) hold promise as biomarkers for HCC detection. The methylation status of DNA in HCC tumour tissues correlates strongly with blood ctDNA, suggesting the feasibility of non‐invasive HCC diagnosis and monitoring using ctDNA‐based tumour‐specific genetic and epigenetic alterations.^113^ Studies on ctDNA methylation biomarkers are categorised into three types: the number of methylation sites, expression of methylation sites and detection of 5‐hydroxymethylcytosines (5hmCs).^114^ The first two have been the focus in the past; however, there has been increasing attention on detecting 5hmC as a third form of epigenetic regulation. This modification occurs on CpG islands and can induce changes in DNA conformation, chromosome structure and DNA‐protein binding.^115^, ^116^ Unlike DNA methylation, where 5mCs is present, 5mCs can be converted into 5hmCs through the enzyme 5mC hydroxylase, leading to mammalian demethylation changes. Reduced levels of 5hmCs in ctDNA have been observed in early HCC patients, suggesting its potential as a useful diagnostic biomarker for early HCC.^117^ Angeli‐Pahim et al. developed a ctDNA methylation score system to evaluate changes in tumour burden without needing a biopsy in HCC.^118^ Zhang et al. identified several hypermethylated genes on plasma ctDNA, including *DBX2, RGS10, ST8SIA6, TGR5, MT1* *M, MT1G, RUNX, THY1, INK4A, VIM, FBLN1* and *SEPT9*, which could serve as potential diagnostic biomarkers for HCC.^114^, ^119^

Assessing cfDNA promoter methylation patterns can help identify high‐risk populations and evaluate treatment responses in HCC, including markers such as vimentin and fibulin1.^120^, ^121^ Wang et al. found significantly higher serum levels of overall cfDNA methylation in patients with HCC, compared to healthy individuals.^122^ Zhao et al. proposed a dual‐marker panel for early HCC detection, combining plasma methylated cfDNA of G protein subunit beta 4 (*GNB4*) and *Riplet*.^123^ Lin et al. identified novel urine cfDNA methylation markers for HCC diagnosis,^124^ including general receptor for phosphoinositides 1‐associated scaffold protein (*GRASP), HOXA9, BMP4* and *ECE1*, with significant differences observed between HCC patients and healthy controls. A predictive model was constructed to demonstrate the high diagnostic potential of methylated urine cfDNA for early HCC screening. Kim et al. also developed a circulating cfDNA methylation signature (including lactate dehydrogenase B [LDHB] and Ring finger protein 135 [*RNF135*]) for effective early detection of HCC.^125^

Furthermore, recent articles have delved into the bioinformatics perspective to construct signatures for HCC based on gene DNA methylation data. These signatures aim to predict patient prognosis and identify molecular biomarkers for HCC diagnosis.^126^, ^127^, ^128^, ^129^, ^130^, ^131^, ^132^, ^133^, ^134^ Zheng et al., analyzing DNA methylation histological data from three datasets, pinpointed *ZSCAN1, DNM3*, cyclin‐dependent kinase‐like 2 (*CDKL2*)*, ASCL2, DUOXA1, NKX6‐2, FSCN1, TBX15* and *TMEM240* as significant biomarkers for HCC.^135^ Ma et al. highlighted B‐cell translocation gene 2 (*BTG2*) as a potential prognostic biomarker for Chinese patients with HCC.^127^ Other studies have utilised DNA methylation data to analyse different HCC subtypes, offering insights for clinical diagnosis and prognosis prediction.^7^, ^29^, ^136^, ^137^, ^138^, ^139^, ^140^, ^141^, ^142^, ^143^, ^144^, ^145^ In addition to their roles in clinical diagnosis, treatment and prognosis assessment, DNA methylation biomarkers hold promise for early tumour detection, potentially improving patient survival outcomes. However, while many research findings are confined to laboratory research, few AFP‐like markers have been widely adopted in clinical practice. Future diagnostic approaches may leverage multiple biomarkers to enhance HCC diagnosis accuracy, predictive sensitivity and specificity.

## 5 MECHANISMS RELATED TO HCC MALIGNANT PROGRESSION REGULATED BY DNA METHYLATION

### 5.1 Relationships between DNA methylation and HCC proliferation, apoptosis and angiogenesis

**FIGURE 2:**
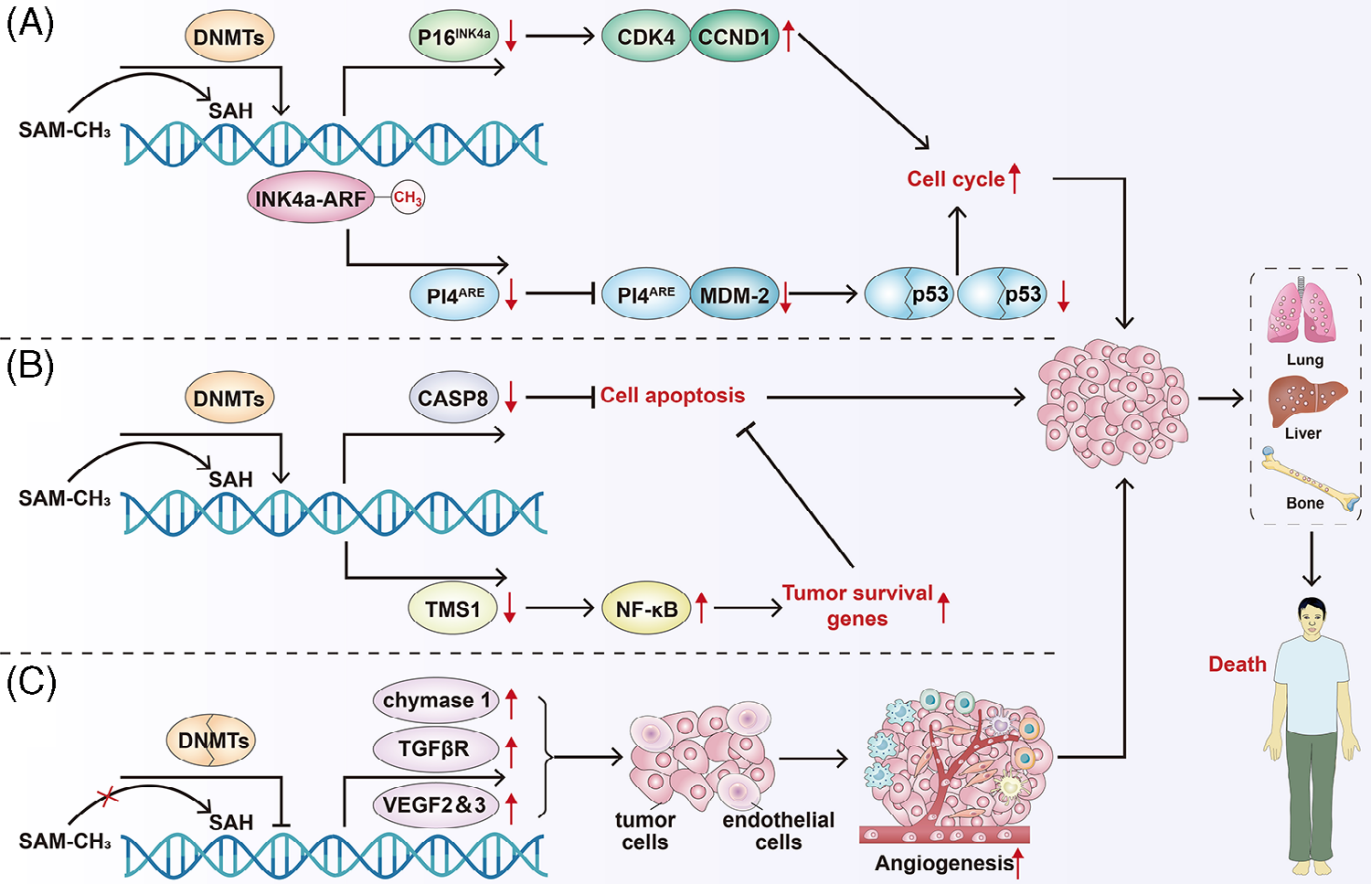
Relationships between DNA methylation and hepatocellular carcinoma (HCC) proliferation, apoptosis and angiogenesis. (A) DNA promoter hypermethylation results in the loss of p16^INK4a^ protein expression, which facilitates the binding of CDK4 and CCND1, then activating the cell cycle. Meanwhile, DNA promoter hypermethylation also reduces p14^ARF^ protein expression, impairs its binding with MDM‐2 and leads to the degradation of the tumour suppressor gene p53, failing to inhibit the cell cycle finally. (B) Hypermethylation of caspase‐8 (*CASP8*) promotor results in silencing of *CASP8* gene expression and inhibiting cell apoptosis. Also, hypermethylation of *TMS1* promoter leads to reduced TMS1 protein expression and ineffective blocking of nuclear factor‐kappa B (NF‐κB) and downstream tumour survival‐related genes’ transcription, then inhibiting cell apoptosis. (C) Hypomethylation of *chymase 1*, *tyrosine kinase nonreceptor 2*, transforming growth factor beta receptor II (*TGFβR*) and vascular endothelial growth factors 2 and 3 (*VEGF2/3*) gene promoters leads to increased expression of these genes. Increased expression of these genes affects the interaction between tumour cells and vascular endothelial cells and contributes to the angiogenesis. Finally, all these above DNA methylation‐related pathways contribute to the metastasis and patients’ death in HCC.

The INK4a‐ARF pathway is crucial to HCC occurrence and progression.^146^ INK4a‐ARK encodes two regulatory proteins of cell cycle (p16^INK4a^ and p14^ARF^), which exert their effects through the Rb‐CDK4 and p53 pathways. P16^INK4a^ inhibits CDK4 binding to CCND1, while p14^ARF^ prevents p53 degradation by binding to MDM‐2 and inducing cell cycle arrest.^147^, ^148^ In HCC, aberrant DNA promoter methylation can result in the loss of p16^INK4a^ protein expression, facilitating the binding of CDK4 and CCND1, activating the cell cycle and ultimately driving malignant progression. Conversely, hypermethylation of the p14^ARF^ gene promoter reduces p14^ARF^ protein expression, impairs its binding with MDM‐2 and leads to the degradation of the tumour suppressor gene p53, failing to inhibit the cell cycle,^149^ thereby promoting the malignant progression. CASP8 promotes apoptosis through cell death receptors and mitochondrial pathways.^150^ Yu et al. reported approximately 72% abnormal hypermethylation of the *CASP8* promoter in HCC,^151^ resulting in silencing of *CASP8* gene expression and enhanced anti‐apoptotic ability of tumour cells. *TMS1/ASC*, another crucial apoptosis‐promoting gene,^152^ inhibits the expression of NF‐κB^153^ and blocks the transcription of tumour‐related survival signal genes. However, the *TMS1* gene promoter's methylation level significantly increases in HCC cells, with up to 80% hypermethylation, leading to reduced TMS1 protein expression and ineffective blocking of tumour survival‐related genes’ transcription.^154^ Treatment of HCC cell lines with the demethylation reagent 5‐azacytidine (5‐AZA) has been shown to restore TMS1 transcription, thereby promoting HCC cell apoptosis.^12^, ^155^ In the tumour microenvironment (TME) of HCC, tumour cells and endothelial cells collaborate to influence angiogenesis,^156^, ^157^, ^158^ which subsequently facilitates tumour invasion and metastasis.^159^, ^160^ DNA methylation changes significantly impact tumour‐related angiogenesis in HCC. Hypomethylation of chymase 1, tyrosine kinase nonreceptor 2, transforming growth factor beta receptor II and vascular endothelial growth factors 2 and 3 gene promoters leads to increased expression of these genes. This dysregulation affects the interaction between tumour cells and vascular endothelial cells, promoting the generation of tumour micro‐vessels and metastasis.^161^, ^162^ Furthermore, Sun et al. found that the expression of collagen triple helix repeat containing‐1 (CTHRC1), a kind of extracellular matrix protein and linked with poor survival outcomes of patients with HCC, was associated with DNA hypomethylation. DNA hypomethylation‐induced CTHRC1 high expression may promote proliferation and angiogenesis and inhibit apoptosis of HCC.^163^ Liu et al. also found that DNA hypomethylation of the insulin‐like growth factor 2 mRNA‐binding protein 3 (IGF2BP3) increased the protein expression of IGF2BP3 and promoted cell proliferation of HCC.^164^ Meanwhile, Zhang et al. found that DNA promoter hypomethylation induced cyclic AMP‐responsive element‐binding protein (CREB)‐regulated transcription coactivator 2 (CRTC2) high expression contributed to the HCC proliferation and indicated higher tumour grade and stage of HCC. Mechanically, hypomethylation‐induced CRTC2 high expression promoted the malignant development of HCC by activating the Wnt pathway and affecting the anti‐tumour immune response.^165^ Yoo et al. reported that DNA hypomethylation‐induced low density lipoprotein (LDL) receptor‐related protein 11 high expression presented a promoting effect on cell proliferation and migration in HCC.^166^ Figure 2 summarises the main correlations between DNA methylation and HCC proliferation, apoptosis and angiogenesis.

### 5.2 Correlations between DNA methylation and adhesion, invasion and metastasis of HCC

**FIGURE 3:**
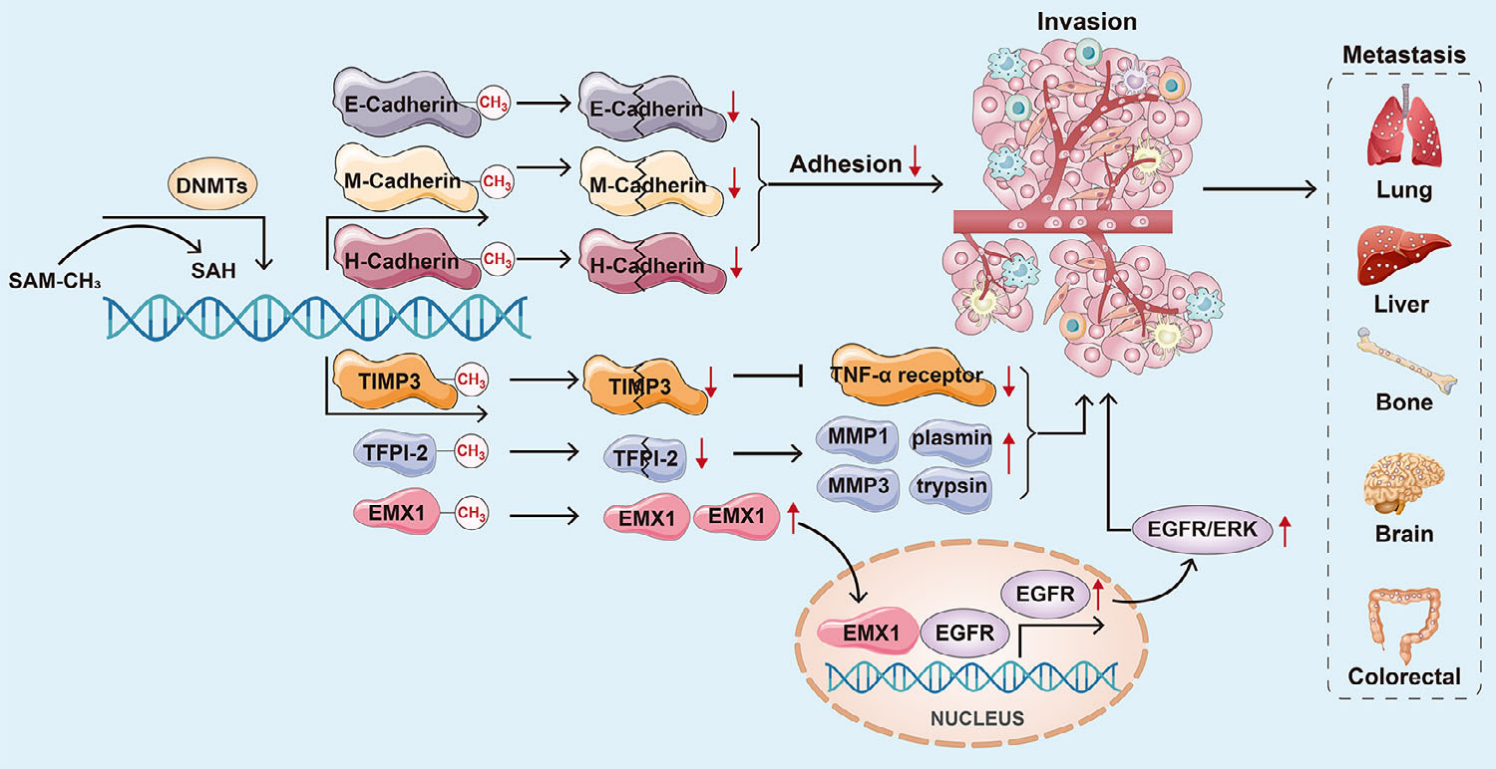
Relationships between DNA methylation and adhesion, invasion and metastasis of HCC. DNA hypermethylation considerably decreases the expression of E‐cadherin, M‐cadherin and H‐cadherin protein, then diminishing the adhesive capacity of tumour cells to a certain extent and enhancing their invasive and metastatic potential. Meanwhile, hypermethylation of *TIMP3* and *TFPI‐2* gene promoter significantly decreases their protein expression, then reduces the expression of TNF‐α receptor in HCC cells and increases the expression of MMP1, MMP3, plasmin and trypsin, finally promoting the invasion and metastasis of HCC cells. Besides, DNA hypermethylation‐activated full‐length empty spiracles homeobox 1 (EMX1) binds to the *EGFR* promoter, enhancing *EGFR* transcription and activating the EGFR‐ERK signalling pathway, significantly promoting HCC invasion and metastasis.

E‐cadherin, a cell membrane glycoprotein, is an adhesion molecule with widespread expression in epithelial cells.^167^, ^168^, ^169^, ^170^ The decreased expression of the E‐cadherin protein disrupts cell adhesion and encourages the invasion and metastasis of cancer cells.^170^, ^171^ Methylation of CpG islands is the primary driver behind the decreased or loss of E‐cadherin protein expression in HCC.^172^ The frequency of hypermethylation in the E‐cadherin gene promoter in HCC ranges from approximately 33% to 67%.^173^, ^174^ Moreover, hypermethylation of the E‐cadherin gene promoter occurs at up to 8% and 46% in dysplastic liver nodules and chronic hepatitis cirrhosis tissues, respectively.^173^ Therefore, the diminished expression of E‐cadherin protein due to DNA promoter hypermethylation is a significant factor in the invasion and metastasis of HCC. Furthermore, DNA hypermethylation reduces the expression of M‐cadherin and H‐cadherin proteins within the same family as E‐cadherin, diminishing the adhesive capacity of tumour cells to a certain extent and enhancing their invasive and metastatic potential.^152^, ^175^ Inhibiting HCC invasion and metastasis is achievable through tissue inhibitors of metalloproteinase‐3 (TIMP‐3).^176^, ^177^, ^178^ Exogenous overexpression of TIMP‐3 considerably inhibits cell invasion and metastasis by stabilising the Tumor necrosis factor‐α (TNF‐α) receptor in HCC cell lines.^177^ However, TIMP‐3 expression significantly decreases in HCC under normal conditions due to CpG island promoter hypermethylation.^179^ Reports indicate that 13%–19% of patients with HCC exhibit hypermethylation of their TIMP‐3 promoter. The abnormal hypermethylation status of the TIMP‐3 promoter significantly promotes HCC cell invasion and metastasis.^180^ Tissue Factor Pathway Inhibitor 2 (TFPI‐2), a Kunitz‐type serine protease inhibitor, impedes the invasion and metastasis ability of tumour cells via inhibiting the activities of Matrix Metallopeptidase 1 (MMP1), Matrix Metallopeptidase 3 (MMP3), plasmin and trypsin.^181^, ^182^ However, in HCC, the frequency of hypermethylation of the TFPI‐2 gene promoter is approximately 47%, leading to a significant decrease in TFPI‐2 protein expression. Moreover, transcriptional reactivation of TFPI‐2 in HCC cell lines has inhibited invasion and metastasis by employing 5‐AZA to demethylate epigenetically silenced *TFPI‐2* gene expression. Wen et al. identified that DNA hypermethylation‐activated full‐length *EMX1* binds to the Epidermal Growth Factor Receptor (*EGFR*) promoter, enhancing EGFR transcription and activating the EGFR‐extracellular signal‐regulated kinase (ERK) signalling pathway, significantly promoting HCC metastasis.^183^ Apart from DNA hypermethylation, DNA hypomethylation has also been associated with the activation of oncogenes such as *c‐Myc*, apoptosis‐inducing factor mitochondria‐associated 2 (*AIFM2*), RAS ribosomal protein lateral stalk subunit p2 (*RPLP2*) and Wiskott–Aldrich syndrome protein family member 2 (*WASF2*).^184^, ^185^, ^186^, ^187^, ^188^, ^189^ DNA hypomethylation has been identified as a factor associated with the development of a more aggressive HCC phenotype, including an increased ability for tumour invasion and metastasis. These findings underscore the significant impact of DNA methylation on the adhesion, invasion and metastasis of HCC. In Figure 3, we summarised the relationships between DNA methylation and adhesion, invasion and metastasis of HCC.

### 5.3 Relationships between DNA methylation and sorafenib resistance in HCC

Sorafenib, a multi‐kinase inhibitor, effectively inhibits proliferation and induces apoptosis in tumour cells. It has emerged as a first‐line therapy for patients with advanced or unresectable HCC. However, sorafenib resistance has become a common challenge for patients with HCC.^190^, ^191^, ^192^ Furthermore, DNA methylation is involved in sorafenib resistance in HCC. Abeni et al. identified 1230 differentially methylated genes in HCC cells, which were treated with sorafenib, compared to control, and observed that oncogenes were more likely to be hypermethylated, while tumour suppressor genes were more likely to be hypomethylated upon the treatment of sorafenib. This result indicates that failure to hypermethylate oncogenes and hypomethylate tumour suppressor genes upon sorafenib treatment may lead to resistance,^193^ although this hypothesis requires further testing.

**FIGURE 4:**
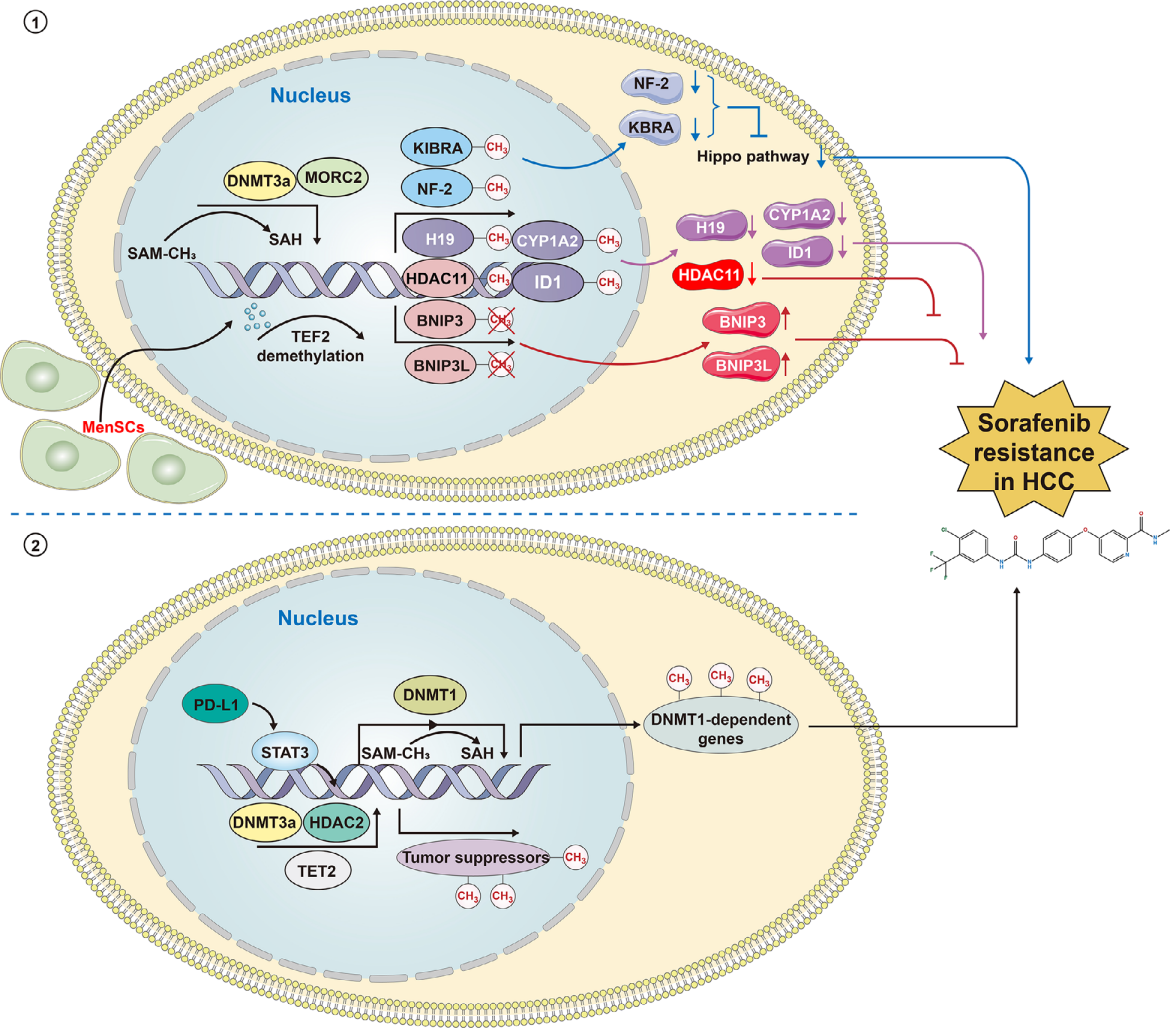
Relationships between DNA methylation and sorafenib resistance in HCC. DNA methylation is obviously involved in sorafenib resistance for patients with HCC. Microrchidia 2 (MORC2) interacts with DNA methyltransferase 3a (DNMT3A) at the promoters of neurofibromin 2 (*NF2*) and kidney and brain protein (*KIBRA*) genes, leading to DNA hypermethylation and transcription inhibition. The expression silence of NF2 and *KIBRA* significantly inactivates the Hippo pathway, thereby causing sorafenib resistance. Meanwhile, DNA hypermethylation also significantly reduces the expression of BCL2 interacting protein 3 (BNIP3) and BCL2/adenovirus E1B 19 kDa interacting protein 3‐like (BNIP3L). However, menstrual blood‐derived stem cells (MenSCs) effectively upregulate the expression of BNIP3 and BNIP3L by mediating significant DNA promoter demethylation through ten‐eleven translocation methylcytosine dioxygenase 2 (TET2), thereby reversing sorafenib resistance in HCC cells. Besides, DNA hypermethylation of long non‐coding RNA (LncRNA) *H19*, *HDAC11*, cytochrome P450 1A2 (*CYP1A2*) and inhibitor of DNA binding 1 (*ID1*)‐induced transcription inhibition also affect sorafenib resistance of HCC. Simultaneously, in sorafenib‐resistant HCC cells, PD‐L1 activates the expression of DNA methyltransferase 1 (DNMT1) via STAT3 and induces the DNA methylation of downstream genes. Furthermore, DNMT3A‐TET2 crosstalk increases the level of promotor DNA methylation of tumour suppressors through a corepressor complex with HDAC2 followed by the decreased expression of related genes, then resulting sorafenib resistance in HCC. These findings demonstrate that DNA methylation plays a significant role in sorafenib resistance. Targeting DNA methylation‐related genes may effectively reverse sorafenib resistance in HCC.

Wang et al. identified the microrchidia 2 (MORC2)‐neurofibromin 2 (NF2)/kidney and brain protein (KIBRA) axis as critical for sorafenib resistance in HCC.^194^ MORC2 interacts with DNMT3A at the promoters of *NF2* and *KIBRA* genes, leading to DNA hypermethylation and transcription inhibition. Since NF2 and KIBRA are critical targets of MORC2 in inducing Hippo signalling activation,^195^, ^196^, ^197^, ^198^ DNA hypermethylation of *NF2* and *KIBRA* significantly inactivates the Hippo pathway, thereby causing sorafenib resistance.^194^, ^199^, ^200^ The LncRNA H19 is significantly downregulated in HCC tissues. LncRNA 19 effectively inhibits the malignant development of HCC. Moreover, the expression and DNA methylation levels of the *H19* gene differ significantly between sorafenib‐sensitive and sorafenib‐resistant cell lines. Overexpression of LncRNA H19 significantly sensitises sorafenib‐resistant HCC cells by inhibiting cell proliferation upon sorafenib treatment.^201^ LncRNA H19 may serve as a potential biomarker to reverse sorafenib resistance in HCC. Zhou et al. observed that promoter DNA methylation significantly reduces tumour suppressor genes BCL2 interacting protein 3 (*BNIP3*) and BCL2/adenovirus E1B 19 kDa interacting protein 3‐like (*BNIP3L*) expression in sorafenib‐resistant HCC cells. Furthermore, human menstrual blood‐derived stem cells effectively upregulate the expression of BNIP3 and BNIP3L by mediating significant DNA promoter demethylation through ten‐eleven translocation methylcytosine dioxygenase 2 (*TET2*), thereby reversing sorafenib resistance in HCC cells.^202^ Liu et al. discovered that in sorafenib‐resistant HCC cells, programmed death‐ligand 1 (PD‐L1) activates the expression of DNMT1 by activating the Signal Transducer And Activator Of Transcription 3 (STAT3). Silencing PD‐L1 promotes DNMT1‐dependent DNA demethylation, reversing sorafenib resistance.^203^ Mo et al. identified the high expression of ten‐eleven translocation protein 1 (TET1) in sorafenib‐resistant HCC cells and silence of TET1 could significantly alleviate sorafenib resistance of HCC. Furthermore, mechanistic analysis found that TET1 could regulate the DNA promoter methylation of DNA repair genes by interacting with yes‐associated protein 1 in sorafenib‐resistant HCC cells. Using TET1 inhibitor Bobcat339 may increase the DNA methylation level of DNA repair‐related genes and inhibit their protein expression, then reverse sorafenib resistance in HCC.^204^ Cheng et al. identified the interaction of *DNMT3A* and *TET2* as an actionable mechanism of sorafenib resistance in HCC cells and HCC stem cell‐like cells. Upregulation of *DNMT3A* and *TET2* leads to increased production of 5mC and 5hmC, resulting in faster proliferation and metastasis of sorafenib‐resistant HCC cells. Mechanistically, DNMT3A‐TET2 crosstalk increases the level of promotor DNA methylation of tumour suppressors (P15, SOCS2) through a corepressor complex with HDAC2 followed by the decreased expression of related genes. Therefore, either genetic silence or pharmacological suppression of DNMT3A or TET2 effectively restored the sorafenib sensitivity of HCC.^205^ Wang et al. observed significantly higher expression of *HDAC11* in sorafenib‐resistant SMMC7721 HCC cells. Silencing *HDAC11* reverses sorafenib resistance effectively. Further studies found markedly reduced DNA methylation levels of *HDAC11* in HCC tissues. Treatment with the 5‐AZA decreased the methylation level of *HDAC11* and facilitated its protein expression, contributing to sorafenib resistance in HCC cells.^206^ However, Gailhouste et al. demonstrated that an epigenetic regimen with non‐cytotoxic doses of 5‐AZA, a demethylating compound, significantly improves sorafenib response in sorafenib‐resistant HCC cells.^207^ The above results indicate that different doses of 5‐AZA may have different effects on sorafenib resistance. Galle et al. clinically validated that changes in DNA promoter methylation drive epithelial‐to‐mesenchymal transition‐mediated sorafenib resistance in advanced HCC. Specifically, they developed a capture‐based protocol to investigate DNA methylation in low amounts of ctDNA. By analyzing the methylation status of ctDNA in liquid biopsies, they identified that DNA methylation changes induce epithelial‐to‐mesenchymal transition, significantly contributing to sorafenib resistance.^208^ Cytochrome P450 1A2 (CYP1A2) is a known tumour suppressor. Zhang et al. found that DNMT3A‐induced hypermethylation of *CYP1A2* may contribute to sorafenib resistance in HCC. Treatment with epigenetic drugs such as decitabine and trichostatin A restored the expression of CYP1A2, enhancing the sorafenib sensitivity for HCC.^209^ Meng et al. discovered that inhibitor of DNA binding 1 was regulated by DNMT3b and exhibited DNA hypermethylation status in sorafenib‐resistant HCC cells. Treatment with 5‐AZA significantly upregulated the expression of ID1 and reversed sorafenib resistance in HCC.^210^ All these findings demonstrate that DNA methylation plays a significant role in sorafenib resistance. Targeting DNA methylation‐related genes may effectively reverse sorafenib resistance in HCC. Figure 4 summarises the relationships between DNA methylation and sorafenib resistance in HCC.

### 5.4 Relationships between DNA methylation and DNA damage repair ability in HCC

The mismatch repair system (MMR) is crucial in DNA replication mechanisms. Defects in MMR can lead to microsatellite instability in tumour cells.^211^, ^212^, ^213^ In HCC, the promoters of the MMR‐related genes exhibit relatively high methylation frequencies. The methylation frequencies of *hMLH1*, *hMLH2* and *hMLH3* promoters are approximately 5%–13%, 68% and 75%, respectively. Moreover, a high methylation frequency of MMR genes is also observed in patients with liver cirrhosis, with 55% and 70% methylation frequencies for *hMLH2* and *hMLH3* gene promoters, respectively.^214^ *MGMT* is a critical DNA repair gene that has high liver activity.^215^ *MGMT* maintains normal cell function by protecting DNA from genetic mutations and cytotoxic drugs. Normal cells can transform into tumours when DNA damage and repair mechanisms malfunction. It is estimated that 22%–39% of HCC cases exhibit abnormal methylation in the *MGMT* gene promoter.^216^ Similarly, the tumour suppressor gene *GSTP1* shields cells from DNA damage caused by mutations and cytotoxic drugs. In HCC, the frequency of hypermethylation of the *GSTP1* gene promoter can range from 41% to 85%.^217^ These findings demonstrated that the hypermethylation status of DNA promoters significantly affects DNA damage repair ability and contribute to the progression of HCC.

### 5.5 Relationships between DNA methylation and liver fibrosis

Liver fibrosis, a standard pathological change associated with HCC, arises from defects in extracellular matrix synthesis and degradation.^218^, ^219^, ^220^ Persistent liver fibrosis is closely associated with HCC proliferation, metastasis and drug resistance.^221^, ^222^, ^223^ Activation of hepatic stellate cell (HSC) is a critical event that could contribute to the development of liver fibrosis, primarily dictated by their DNA methylation status. Methylation at specific DNA sites in activated hematopoietic stem cells (HSCs) can downregulate IκBα expression, transforming HSCs into fibroblast‐like states and contributing to liver fibrosis. Demethylation reagents can reverse this process.^224^ Patched 1 (*PTCH1*) gene promoter methylation is implicated in HSC activation, with methylation inhibitors increasing PTCH1 expression and inhibiting HSC activation. Abnormal methylation in HSCs results in altered CD133, CD44 and Notch1 expression and impaired differentiation of HCC stem cells.^225^, ^226^ Moreover, hypermethylation of the suppressor of cytokine signaling‐3 (*SOCS3*) gene promoter reduces SOCS3 expression, activating STAT3 and upregulating TGFβ‐1 expression, exacerbating liver fibrosis.^227^ DNA hypermethylation in abnormal liver macrophages also contributes to liver fibrosis.^228^ Therefore, DNA methylation abnormalities significantly deepen fibrosis within the liver microenvironment, predisposing individuals to HCC development.

### 5.6 Relationships between DNA methylation and tumour immune escape

**FIGURE 5:**
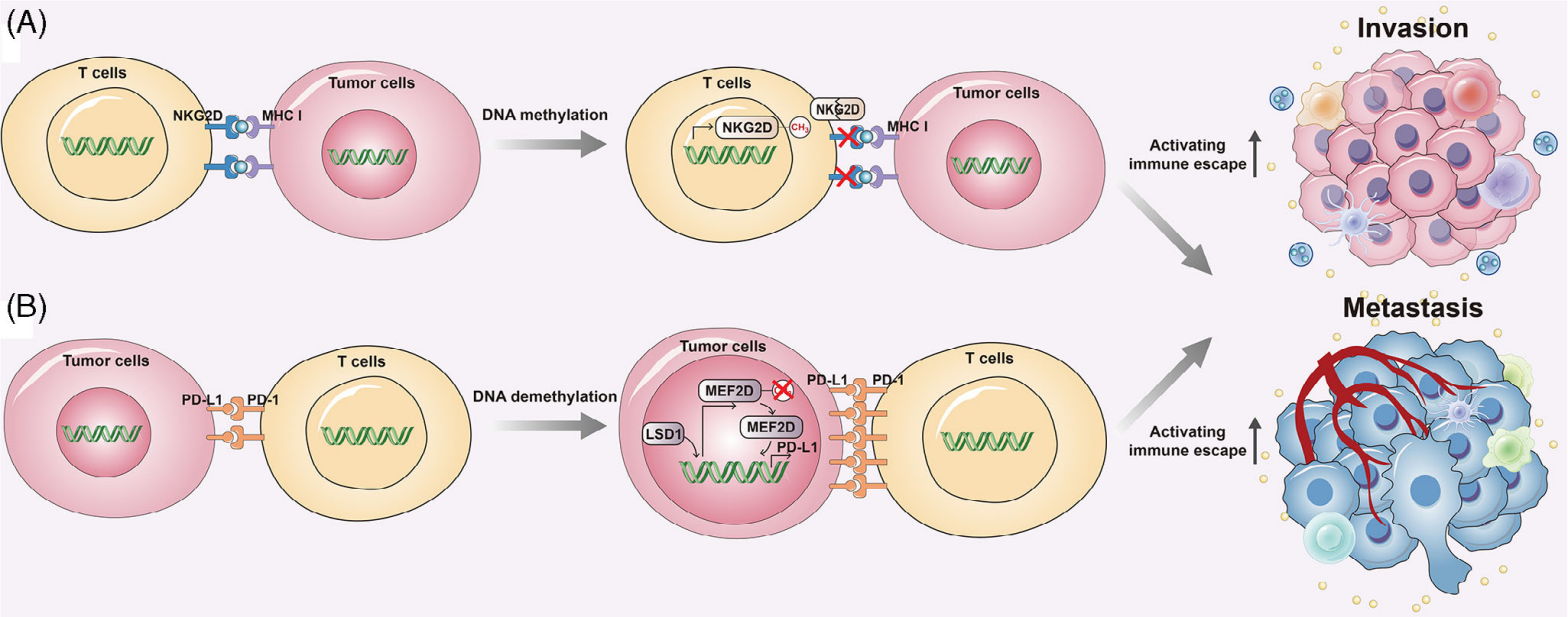
Relationships between DNA methylation and tumour immune escape. (A) In normal condition, natural killer cell receptor group 2D (NKG2D), expressed on T cells, could activate lymphocyte by recognising MHC I‐like ligands expressed on tumour cell, thereby enhancing immune capacity and eliminating tumour cells. However, the expression of NKG2D was regulated by DNA methylation. In HCC, elevated DNA methylation of *NKG2D* gene leads to reduced NKG2D protein expression, resulting in decreased activation of lymphocytes and activating tumour immune escape. (B) DNA demethylation is also involved in the process of tumour immune escape. Lysine‐specific demethylase 1 (LSD1) interacts with myocyte enhancer factor 2D (MEF2D) to decrease its DNA methylation level and increase its protein expression. Then, MEF2D binds to the promoter of *PD‐L1*, activating PD‐L1 expression. Furthermore, PD‐L1 binds with PD‐1, which attenuates the response of HCC cells to T cell‐induced cytotoxic effects, leading to tumour immune escape.

Within the HCC microenvironment, the interplay between immune cells and tumour cells significantly impacts HCC recurrence and metastasis.^159^, ^229^, ^230^ Regulatory T cells (Tregs) are notably enriched in HCC, with their abundance inversely correlated with patient prognosis.^231^ However, abnormal DNA methylation has been observed in Tregs, liver cells and peripheral monocytes among patients with HCC induced by the hepatitis B virus.^232^ Aside from its role as an effective biomarker for HCC detection,^95^ the *NKG2D* receptor has been found to confer protection against tumour invasion. NKG2D, expressed on natural killer cells (NK cells) and T cells, activates lymphocytes by recognising major histocompatibility complex I (MHC I)‐like ligands on tumour cells, thereby enhancing immune capacity and eliminating tumour cells.^233^, ^234^, ^235^, ^236^ Subsequent studies revealed that DNA methylation regulates NKG2D expression, with HCC patients exhibiting significantly higher DNA methylation frequency in the *NKG2D* gene promoter, compared to chronic hepatitis B patients and healthy individuals.^237^ Elevated DNA methylation of the *NKG2D* gene leads to reduced NKG2D protein expression, resulting in decreased activation of lymphocytes and subsequent tumour cell killing.^238^ Furthermore, Wang et al. identified lysine‐specific demethylase 1A as a regulator of PD‐L1 expression through its interaction with myocyte enhancer factor 2D (MEF2D) and acting as a demethylase to significantly decrease its DNA methylation level, and then increase the protein expression of MEF2D. Demethylated MEF2D binds to the *PD‐L1* promoter, activating PD‐L1 expression,^239^ which attenuates the response of HCC cells to T cell‐induced cytotoxic effects, leading to immune escape.^240^, ^241^ Moreover, abnormal methylation of the *RNF39* gene promoter has been associated with immune escape in HCC,^242^, ^243^ as *RNF39* is involved in antigen presentation and processing within the MHC complex. These findings underscore the significant role of DNA methylation‐mediated tumour immune escape in HCC progression. Figure 5 summarises the correlations between DNA methylation and tumour immune escape in HCC.

### 5.7 Relationships between DNA methylation and the activation of HCC tumour stem cells

**FIGURE 6:**
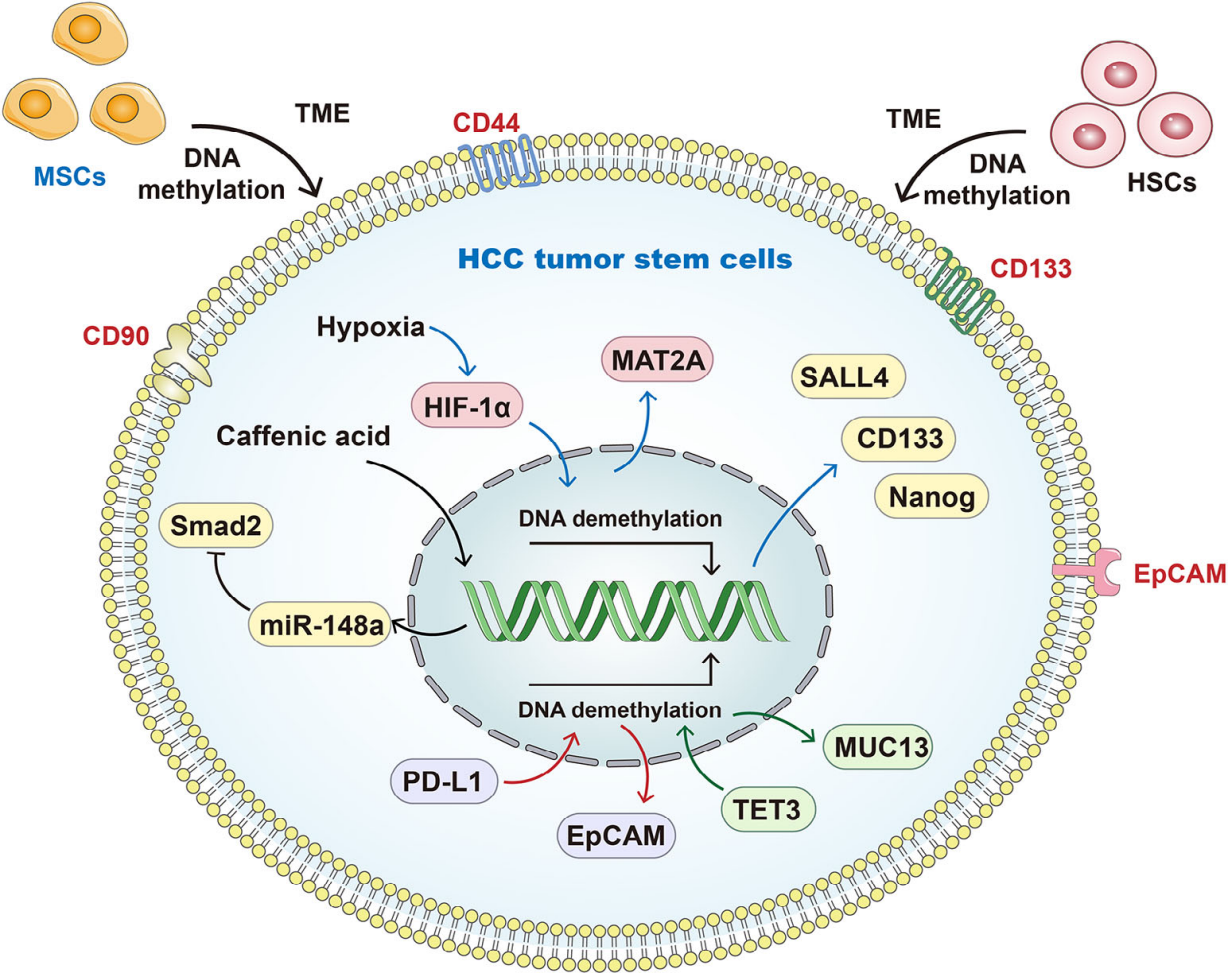
Relationships between DNA methylation and HCC tumour stem cells. CD44, CD90, epithelial cell adhesion molecule (EpCAM) and CD133 are all the markers of tumour stem cells in HCC. Besides, in the aberrant HCC tumour microenvironment (TME), normal mesenchymal stem cells (MSCs) and hematopoietic stem cells (HSCs) cease differentiation into liver cells and instead undergo reverse differentiation into tumour stem cells, a process regulated by DNA methylation. Moreover, hypomethylation of DNA promoters contributes to increased sal‐like 4 (SALL4), CD133 and Nanog expression in tumour stem cells. Concurrently, hypoxia within the TME upregulates the expression of HIF‐1α, leading to DNA demethylation and increased expression of the methionine adenosyltransferase 2A (*MAT2A*) gene. Caffeic acid also elevates DNA methylation levels in stem cells, subsequently downregulating Smad2 expression mediated by miR‐148a. Meanwhile, silencing PD‐L1 significantly induced global DNA hypomethylation and dysregulation of EpCAM expression. At last, TET3‐induced promoter hypomethylation of Mucin 13 (MUC13) boosted its expression in tumour stem cells, thereby enhancing the generation and activation of tumour stem cells characteristics. All these results prove the close relationships between DNA methylation and the activation of HCC tumour stem cells.

Surface biomarkers such as CD44, CD90, epithelial cell adhesion molecule (EpCAM) and CD133 are utilised to identify tumour stem cells in HCC, whose activation profoundly drives the malignant progression of the disease.^244^, ^245^, ^246^ In the aberrant HCC TME, normal mesenchymal stem cells and HSCs cease differentiation into liver cells and instead undergo reverse differentiation into tumour stem cells, a process regulated by DNA methylation. Furthermore, DNA methylation contributes to the silencing of *DNMT1* expression and the acquisition of tumour stem cell features by normal liver cells.^247^ Moreover, hypomethylation of DNA promoters contributes to increased Sal‐like 4 expression in tumour stem cells, a factor strongly linked to unfavourable prognoses in patients with HCC.^248^ Furthermore, DNA methylation regulates the expression of CD133 and Nanog, recognised as stem cell markers.^249^, ^250^ The heightened expression of these markers due to hypomethylation endows tumour cells with a stem cell‐like phenotype, thereby promoting the progress of HCC.^251^, ^252^ Concurrently, hypoxia within the HCC microenvironment upregulates the expression of Hypoxia Inducible Factor 1 Subunit Alpha (HIF‐1α) in stem cells, leading to DNA demethylation of the methionine adenosyltransferase 2A (*MAT2A*) gene. The resultant increased expression of MAT2A protein fosters the proliferation and metastasis of HCC.^253^ Li et al. reported that caffeic acid elevates DNA methylation levels in stem cells, subsequently downregulating Smad2 expression mediated by miR‐148a, ultimately inhibiting HCC progression.^254^ Sukowati et al. demonstrated that silencing *PD‐L1* in aggressive HCC cell lines significantly inhibited DNMT1 expression, accompanied by global DNA hypomethylation and dysregulation of the tumour stem cell marker EpCAM.^255^ Meanwhile, Kong et al. uncovered that TET3 induced promoter hypomethylation of Mucin 13 boosted its expression in HCC cells and tumour stem cells, thereby enhancing the generation and activation of tumour stem cells characteristics.^256^ The regulation mechanism of DNA methylation in tumour stem cells has increasingly become a focal point of research, representing a current hotspot in the field. A comprehensive understanding of such molecular mechanisms holds critical significance for advancing HCC research. Figure 6 summarises the correlations between DNA methylation and the activation of HCC tumour stem cells.

### 5.8 DNA methylation changes related to alcohol, sex and race in HCC

Alcohol consumption has been linked to epigenetic alterations in numerous studies.^257^, ^258^ Chronic alcohol abuse is substantially associated with an elevated risk of HCC, as it facilitates disease progression.^259^, ^260^, ^261^ Simultaneously, epigenetic modifications are pivotal in HCC progression.^262^, ^263^ Consequently, alcohol‐induced epigenetic changes are poised to influence the onset and evolution of HCC. Alcohol can induce aberrant gene methylation in liver cells by disrupting one‐carbon metabolism and suppressing DNMT activity.^258^, ^264^, ^265^ Such abnormal methylation induced by alcohol is increasingly implicated in the pathogenesis of HCC.^258^ The specific mechanisms underlying these processes have been elucidated. Alcohol‐induced inactivation of the methionine adenosyltransferase 1A (*MAT1A*) gene diminishes the activity of methionine adenosyltransferase in the livers of patients with chronic alcoholism. Consequently, this reduction in MAT activity leads to decreased SAM‐CH3 biosynthesis, the primary methyl source in DNA methylation. Subsequently, HCC progression is facilitated by decreased DNA methylation levels and increased expression of related oncogenes.^266^

Distinct prevalence rates of HCC have also been observed between male and female patients^267^, ^268^ and across various ethnic groups.^269^ Given the established significance of DNA methylation in HCC development, the role of sex and race remains unexplored. Hypermethylation of the *p16^INK4A^* promoter enhances early HCC development and is pivotal in transforming normal liver cells into HCC cells. However, the probability of *p16^INK4A^* promoter hypermethylation was considerably higher among men than women (46.9% vs. 27.2%).^270^ Similarly, *CDKL2*, an essential regulator of tumour cell cycle and proliferation, exhibits significantly increased DNA methylation levels and reduced protein expression in HCC. However, female patients show a considerably higher *CDKL2* promoter methylation frequency than male patients (48.0% vs. 37.7%).^271^ *LINE‐1*, a biomarker for diagnosing HCC,^80^ demonstrates significantly higher levels of DNA hypomethylation at the *LINE‐1* gene promoter in men, compared to women (59% vs. 26%).^272^ Further analysis by Liu et al. revealed that among seven HCC‐related genes, including *APC*, *E‐cadherin*, *DKK*, *WIF1*, *RUNX3*, *SFRP1* and *DLC1*, the proportion of male patients with hypermethylation in more than three genes was higher than that of female patients (65% vs. 41%).^273^

Furthermore, ethnically related DNA methylation changes in HCC have been investigated. Studies have indicated that *CDH1*, which influences tumour cell adhesion and invasion through DNA promoter methylation, is more prevalent in Australians compared to South Africans (30% vs 13%).^274^ DNA methylation also controls *GSTP1*, a tumour suppressor gene whose promoter is more frequently hypermethylated in the Asian population than in the non‐Hispanic White population (hazard ratio: 34.7 vs. 8.9).^275^ These findings underscore the importance of considering gender and race in analyzing the impact of DNA methylation on HCC.

## 6 DNA METHYLTRANSFERASES: NEW TARGETS FOR HCC TREATMENT

DNA methyltransferase catalyses the methylation reaction, resulting in methylated DNA. This group includes DNMT1, DNMT3a and DNMT3b, with DNMT1 responsible for maintaining DNA methylation, while DNMT3a and DNMT3b act as de novo DNA methyltransferases.^276^, ^277^, ^278^, ^279^ Elevated DNMT activity has been observed in HCC,^280^, ^281^, ^282^ with a significant increase in the expression of DNMT1, DNMT3a and DNMT3b observed in liver cirrhosis and HCC tissues.^283^, ^284^, ^285^ Furthermore, the prognosis of HCC patients is closely linked to DNA methyltransferase expression.^286^, ^287^ Several studies have elucidated how DNA methyltransferase regulates epigenetic changes contributing to HCC development.

**FIGURE 7:**
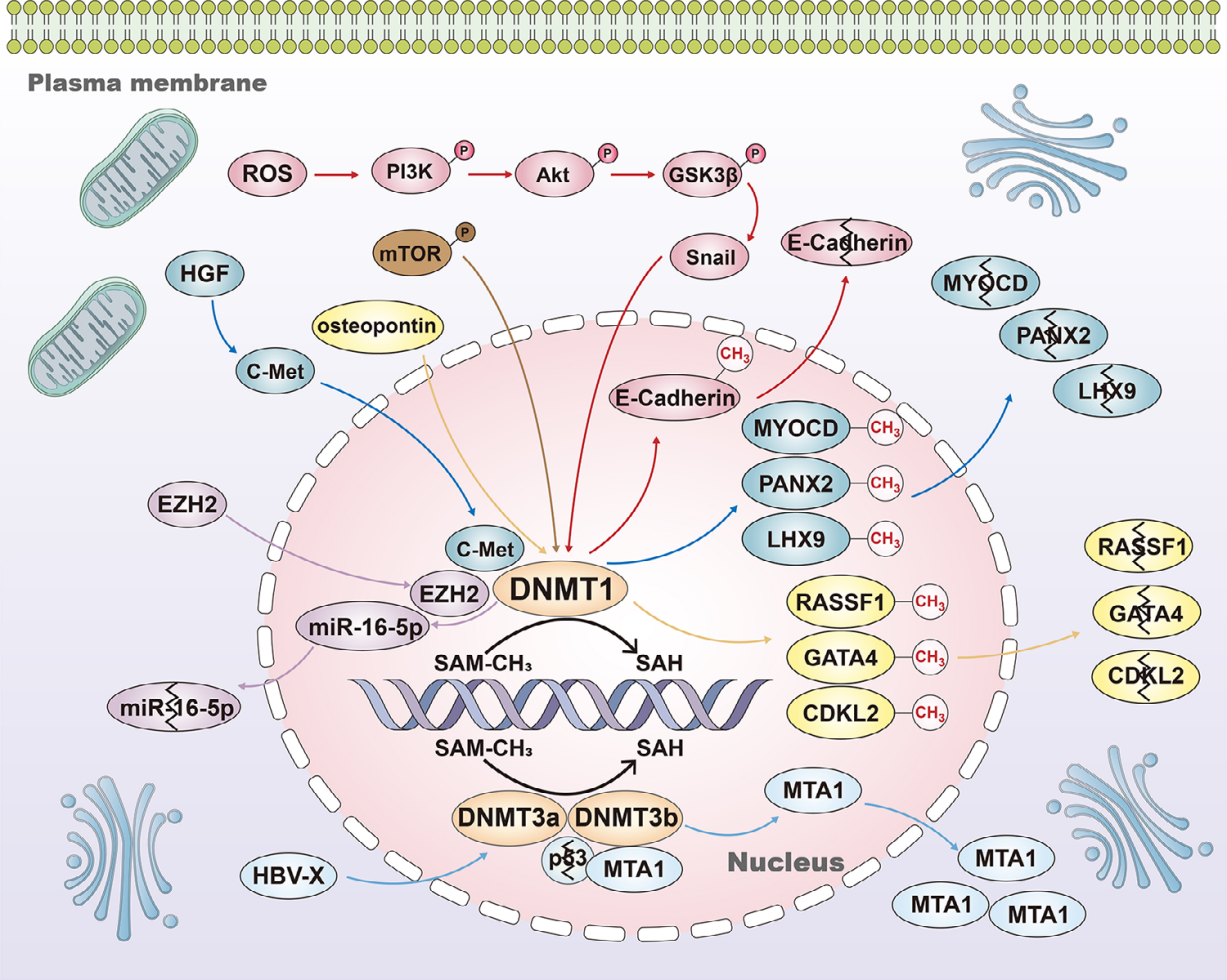
The pivotal role of DNA methyltransferase in DNA methylation and HCC progression. First, osteopontin increases the expression of DNMT1 in HCC stem cells, then induces the DNA methylation of *RASSF1A*, *GATA4* and cyclin‐dependent kinase‐like 2 (*CDKL2*) gene promoters and decreases their protein expressions to activate HCC tumour stem cells. Besides, under the influence of the hepatocyte growth factor (HGF)/c‐Met axis, upregulated DNMT1 expression leads to DNA hypermethylation of tumour suppressor genes myocardin (*MYOCD*), pannexin 2 (*PANX2*) and LIM homeobox 9 (*LHX9*), decreasing related protein expression and promoting the malignant progression of HCC. Reactive oxygen species (ROS)‐induced high expression of Snail via activation of the PI3K/AKT/GSK3β pathway also contributes to the methylation of *E‐cadherin* promoters by recruiting DNMT1, which lowers E‐cadherin expression and ultimately increases invasion and metastasis of HCC. Meanwhile, zeste homolog 2 (EZH2) recruits DNMT1 to miR‐16‐5p DNA promoter, inhibiting the transcription of miR‐16‐5p through DNA methylation, thereby regulating HCC progression. mTOR signalling activation also promotes DNA methylation by inducing DNMT1 translation, contributing to the malignant development of HCC. At last, the HBV‐X protein can induce methylation of CpG island in the *MAT1* promoter, promote the transcription of the *MAT1* gene by recruiting DNMT3a and DNMT3b, then interfering with the DNA binding of p53 in a specific DNA region and inhibiting p53 transcription. Subsequently, MAT1 significantly promotes the malignant progression of HCC. These studies demonstrate that DNA methyltransferase directly affects the DNA methylation level of various essential genes, thereby contributing to the rapid progression of HCC.

In CD133^+^/CD44^+^ HCC stem cells, osteopontin (*OPN*) enhances the metastatic ability by regulating DNA methylation. Silencing *OPN* inhibits DNMT1 expression in HCC stem cells, impairing their spheroid‐forming abilities. The inhibition of DNMT1 expression decreases the DNA methylation level of tumour suppressor genes such as *RASSF1A*, GATA binding protein 4 (*GATA4*) and *CDKL2*.^288^ Hepatocyte growth factor (HGF) and its receptor c‐Met play significant roles in HCC proliferation and invasion,^289^, ^290^, ^291^ with upregulated expression of epigenetically modified c‐Met closely linked to HCC invasion and metastasis.^292^, ^293^, ^294^ Under the influence of the HGF/c‐Met axis, upregulated DNMT1 expression leads to DNA hypermethylation of tumour suppressor genes such as myocardin (*MYOCD*), pannexin 2 (*PANX2*) and LIM homeobox 9 (*LHX9*), promoting the malignant progression of HCC.^295^ Tumour cell invasion and metastasis are also induced by reactive oxygen species,^296^, ^297^, ^298^ which upregulates Snail expression via activation of the phosphoinositide 3‐kinase (PI3K)/AKT/glycogen synthase kinase 3 beta (GSK3β) pathway in HCC. In this context, Snail induces the methylation of *E‐cadherin* promoters by recruiting DNMT1, which lowers E‐cadherin expression and ultimately increases invasion and metastasis of HCC.^299^ Zhang et al. reported that DNMT1 is recruited to miR‐16‐5p DNA promoter via enhancer of zeste homolog 2 (*EZH2)*, inhibiting the transcription of miR‐16‐5p through DNA methylation, thereby regulating HCC progression.^300^ Meanwhile, Chen et al. discovered that mammalian target of rapamycin (mTOR) signalling activation promotes DNA methylation by inducing DNMT1 translation, contributing to the malignant development of HCC. Their study suggested that the combined targeting of mTOR and DNMT1 could lead to a more effective tumour‐killing effect in HCC.^301^ Furthermore, Oh et al. found that increased DNMT3b expression is related to shorter survival time in HCC patients.^302^ In hepatitis B‐related HCC, the Hepatitis B virus‐X (HBV‐X) protein can induce methylation of CpG island in the metastasis‐associated protein 1 (*MTA1*) promoter and promote the transcription of the *MTA1* gene by recruiting DNMT3a and DNMT3b, then interfering with the DNA binding of p53 in a specific DNA region and inhibiting *p53* transcription.^303^ Subsequently, *MTA1* significantly promotes the proliferation, invasion, migration and angiogenesis of HCC.^304^ This result indicates that DNMT3a and DNMT3b induced‐DNA methylation may also promote the expression of related genes; however, the specific molecular mechanism still needs to be explored deeply. In summary, these studies demonstrate that DNA methyltransferase directly affects the DNA methylation level of various essential genes, thereby regulating the malignant progression of HCC. Given the pivotal role of DNA methyltransferase in DNA methylation and HCC progression (Figure 7), researchers have developed DNMTi to target the above pathways.

DNMTi encompasses both nucleoside and non‐nucleoside‐derived inhibitors.^11^ Nucleoside‐derived inhibitors include 5‐AZA, decitabine and zebularine. Among these, 5‐AZA and decitabine were approved for clinical use in 2004 and 2006, respectively.^305^, ^306^ 5‐AZA selectively inhibits DNA methylation by targeting DNA methyltransferase, reducing DNMT expression and DNA methylation levels at low concentrations.^307^ Conversely, high concentrations of 5‐AZA do not yield a corresponding effect. Lower concentrations of 5‐AZA significantly inhibit HCC cell proliferation, invasion, migration and other malignant biological activities.^308^, ^309^ When combined with monoclonal antibodies targeting PD‐L1, 5‐AZA increases the ratio of cytotoxic T lymphocytes in the TME, thereby impeding tumour progression by enhancing lymphocytes’ tumour‐killing capacity.^310^ Moreover, lower concentrations of 5‐AZA can decrease the DNA methylation level of the growth arrest and DNA damage‐inducible 45 beta (*GADD45B*) gene, thereby enhancing the sensitivity of HCC cell lines SMMC‐7721 and Hep‐3B to the chemotherapy drug, cisplatin, consequently amplifying the tumour‐killing effect of chemotherapy drugs.^311^ Furthermore, 5‐AZA in HCC can upregulate the expression of microRNA‐23b‐3p, which leads to the inhibition of cell proliferation and migration.^312^ However, Miranda‐Roblero et al. found another interesting phenomenon. In the presence of 5‐AZA, pirfenidone (PFD) treatment could further inhibit tumour cell proliferation, decrease the expression of Glipican‐3, β‐catenin and c‐Myc and activate lipid metabolism in HCC. However, PFD treatment augmented DNMT1 and DNMT3a expression and restored the global methylation level in HCC. This finding suggested that the role of DNA methylation level and DNMTi in the progression of HCC may be very complex, which may have different effects in different conditions and need to be further deeply explored in the future.^313^ Meanwhile, decitabine inhibits DNA methylation and restores the expression of tumour suppressor genes by reducing DNA methylation levels. Decitabine can directly induce DNA hypomethylation following phosphorylation and increase the apoptosis rate.^314^ Researchers have observed that low concentrations of decitabine in HCC can upregulate the expression of cysteine‐rich zinc‐binding domain homeobox domain 6,^315^ GRASP,^316^ apolipoprotein A1^317^ and delta‐like 3^318^ through DNA demethylation, thereby inhibiting proliferation of HCC, while inducing apoptosis. Zebularine, a newer DNMT inhibitor, exhibits better selectivity and stability than 5‐AZA and decitabine, with fewer side effects.^314^, ^319^, ^320^ Zebularine effectively reduces DNMT1 expression, inhibits DNA methylation levels, restores the expression of relevant tumour suppressor genes and further inhibits the expression of related oncogenes.^247^ Zebularine's anti‐tumour effects are enhanced with Janus kinase/signal transducer and activator of transcription (JAK/STAT) or RAS inhibitors. Moreover, long‐term oral administration of zebularine can induce tissue‐specific demethylation reactions, indicating its potential as a promising DNMT inhibitor.^43^, ^321^

Non‐nucleoside inhibitors include procainamide, procaine, SGI‐1027, (−)‐epigallocatechin gallate (EGCG), hydralazine, RG108 and so forth. Originally used as anaesthetics and antiepileptic drugs,^322^ procainamide and procaine were later found to act as demethylating agents, reactivating the tumour suppressor genes to inhibit tumour cell proliferation, with procainamide specifically targeting DNMT1.^323^ However, the clinical use of these two medicines is limited by their potential adverse effects on cardiac functions.^324^ SGI‐1027, a novel DNMT inhibitor with high lipid solubility, reduces DNA methylation levels by promoting the degradation of DNMT1, thereby restoring the expression of tumour suppressor genes.^325^ At present, SGI‐1027 was reported to decrease the methylation level of several apoptosis‐related genes (Bcl‐2 and Bax), although the precise mechanism of action remains unclear.^326^ EGCG, a polyphenol found in tea, has demonstrated the ability to promote apoptosis (programmed cell death) in malignant cells.^327^, ^328^, ^329^ In HCC, EGCG reduces DNA methylation levels by inhibiting the activity of DNMTs, decreasing mitochondrial membrane potential and inducing cell cycle arrest.^330^ Moreover, EGCG may improve the clinical outcomes of individuals with HCC.^331^ Due to its potent anti‐tumour properties and natural origin, EGCG has not been associated with severe adverse reactions, making it a promising candidate for clinical tumour treatment. As for hydralazine, a DNA hypomethylating agent, Liu et al. found that the combination of routine chemotherapy and hydralazine could significantly prolong the progression‐free survival of HCC patients, compared to chemotherapy alone.^332^

DNMTi are frequently combined with other medications in clinical practice. Examples include 5‐AZA with vitamin C^333^ and SGI‐110 with oxaliplatin.^334^ The combined therapy shows promising anti‐HCC effects. However, the lack of specificity, cytotoxicity and potential adverse reactions of DNMTi limit their long‐term use. Future research efforts may focus on improving the specificity of these drugs, reducing adverse reactions and expanding their clinical application. The available DNMTi, which have achieved excellent anti‐HCC effects, are summarised in Table 4.

**TABLE 4:** A summary of available DNA methyltransferase inhibitors which have achieved excellent anti‐HCC effects.

Classification	Name	Chemical structure	Function	Reference
Nucleoside‐derived inhibitors	5‐Azacytidine (5‐AZA)	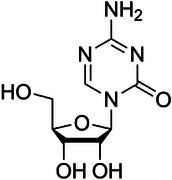	Inhibiting DNA methylation by specifically targeting DNA methyltransferase; low concentration of 5‐AZA significantly inhibits HCC cell proliferation, invasion, migration and other malignant biological activities; when used in combination with monoclonal antibodies of PD‐L1, 5‐AZA increases the ratio of cytotoxic T lymphocytes in the tumour microenvironment and prevents tumour progression by increasing lymphocytes’ tumour‐killing capacity	^307^, ^308^, ^309^, ^310^, ^311^, ^312^, ^313^
	Decitabine	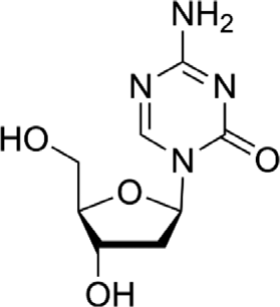	Inhibiting DNA methylation and reactivating the expression of tumour suppressor genes by reducing DNA methylation, then inhibiting the proliferation, invasion, migration and other malignant biological behaviours of HCC and inducing apoptosis	^314^, ^315^, ^316^, ^317^, ^318^
	Zebularine	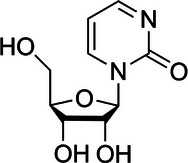	Having better selectivity and stability than 5‐AZA and decitabine and fewer side effects; effectively reducing the expression of DNA methyltransferase 1 (DNMT1), inhibited the level of DNA methylation, restoring the expression of relevant tumour suppressor genes and further inhibiting the expression of related oncogenes	^247^, ^314^, ^319^, ^320^
Non‐nucleoside‐derived inhibitors	Procainamide	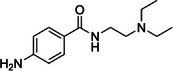	Specifically targeting DNMT1 and acting as demethylated drugs to reactivate the expression of tumour suppressor genes to inhibit tumour cell proliferation	^323^
	Procaine	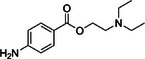	Acting as demethylated drugs to reactivate the expression of tumour suppressor genes to inhibit tumour cell proliferation.	^323^
	SGI‐1027	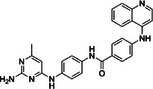	A new DNMTs inhibitor with high‐fat solubility, which can reduce the level of DNA methylation by promoting the degradation of DNMT1, and then restore the expression of the tumour suppressor genes	^325^, ^326^
	(−)‐Epigallocatechin gallate	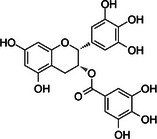	A natural compound, reducing the level of DNA methylation by inhibiting the activity of DNMTs, reducing the mitochondrial membrane potential and promoting cell cycle arrest in the G0‐G1 phase	^327^, ^328^, ^329^, ^330^, ^331^
	Hydralazine	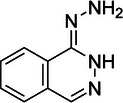	A DNA hypomethylating agent, a combination of routine chemotherapy and hydralazine could significantly prolong the progression‐free survival of patients with HCC	^332^

## 7 THE APPLICATION OF ARTIFICIAL INTELLIGENCE (AI) MODEL TO PREDICT THE CLINICAL OUTCOMES OF HCC PATIENTS BASED ON THE DNA METHYLATION PROFILES

At present, the application of an AI model to predict the clinical outcomes of tumour patients based on the DNA methylation profiles has been a research hotspot. Many researchers have obtained meaningful results. First, Ogunleye et al. applied machine learning (ML) to predict the response to gemcitabine for patients with pancreatic adenocarcinoma (PAAD) partly based on combining 12 DNA methylation probes. Totally, they constructed seven ML models that were predictive and can be used to evaluate other PAAD patients’ treatment response to gemcitabine.^335^ Then, Bomane et al. also applied an AI model to predict the response to paclitaxel in patients with breast cancer based on DNA methylation profiles.^336^ After evaluating 10 ML algorithms on the molecular profiles of breast cancer patients and the results of 60 classifiers on the same patients, they found DNA methylation and miRNA profiles may be the most informative to construct the prediction signatures. Combining the two profiles, first, they built a specific XGBoost classifier that was based on CpG island methylation information and molecular factors to predict the response to paclitaxel. Then, a CpG site methylation‐based decision tree (DT) that combined only two of the selected CpG sites and a microRNA expression‐based DT including only four of the analysed microRNAs were also applied to predict the response to paclitaxel for breast cancer patients. Moreover, the prediction abilities of these signatures were all validated.

In this review, we mainly want to explore and summarise the role of DNA methylation in HCC. Therefore, we further focus on the application of AI models to predict the clinical outcomes of HCC patients based on the DNA methylation profiles. Some studies have obtained corresponding results.^39^, ^337^, ^338^, ^339^, ^340^, ^341^, ^342^, ^343^, ^344^ Kaur et al. first used ML algorithms to separate HCC into early stage or late stage based on 21 CpG methylation sites and 30 RNA transcripts, which had a prediction accuracy of more than 78% on the validation group. Meanwhile, another prediction signature was also built based on five CpG sites and five RNA transcripts and can differentiate HCC and normal samples along with a prediction accuracy of more than 96%.^338^ Furthermore, Chaudhary et al. integrated RNA sequencing data, miRNA sequencing and DNA methylation data to develop a deep learning (DL)‐based model, which can be applied to predict the survival outcomes of patients. The DL‐based model could separate HCC patients into high‐risk and low‐risk groups with significant survival differences (*p *< .001) and a good model fitness. In addition, they also found the positive relationships between the high‐risk group and the high frequency of TP53 inactivation mutations, high expression of tumour biomarkers (KRT19, EpCAM and BIRC5) and the activated pro‐tumour AKT and Wnt pathway.^339^ Similarly, Huang et al. constructed a model based on the bidirectional deep neural networks, which also integrated DNA methylation and mRNA expression data and categorised HCC patients into poor and good prognosis groups. This model also possesses excellent prognostic prediction ability.^340^ At the same time, Bedon et al. built a novel DNA methylation‐related ML model to predict the risk of HCC development.^344^ In this model, they generated a novel signature containing four methylation probes that could categorise patients into high and low risk subgroups and predict the HCC progression. Besides, Deng et al. combined the methylation sequencing and neural networks analysis to identify cfDNA that may carry aberrant DNA methylation. The AI models offered a novel and non‐invasive early detection method for HCC patients. Specifically, they developed a novel DNA methylation detection approach called NEEM‐seq, which has features of low DNA damage and high fidelity, and a read‐level neural detection model called DeepTrace, which can effectively identify sequencing reads derived from HCC. By combining these two AI models, they can easily detect early HCC with surprisingly high sensitivity and specificity (even more than 90%).^39^ Similar to this study, Li et al. developed a similar AI model called DISMIR, which could achieve HCC early detection by analyzing the methylation information of plasma cfDNA and presented high accuracy even at a quite low sequencing depth.^343^ There are also other similar AI models that utilised the DNA methylation information of cfDNA to achieve the early detection of HCC.^345^, ^346^ In short, compared with using a single biomarker of DNA methylation, all these DNA methylation‐related AI models exhibited a higher prediction ability of clinical outcomes for patients with HCC.

## 8 CONCLUSION

This review provides a comprehensive and updated analysis of the mechanisms and detection methods for DNA methylation in HCC, DNA methylation biomarkers for diagnosis, treatment and prognostic assessment of HCC and therapeutic drugs targeting DNMTs. DNA methylation is crucial in tumour progression, advancing tumour diagnosis, prevention and treatment strategies. DNA methylation biomarkers hold promise for assisting in the diagnosis, treatment and prognosis assessment of HCC, offering potential targets for clinical intervention. Several clinical trials involving DNMTs are also underway. However, challenges persist, including the cytotoxicity of DNMT inhibitors, adverse reactions and the lack of efficacy in certain tumours. Prioritising the enhancement of patient quality of life and survival through developing suitable drugs remains imperative. Future endeavours should aim to further analyse the DNA methylation mechanism in HCC, clarify the action mechanism of DNMT inhibitors, mitigate adverse effects, develop novel diagnostic and treatment modalities, pave the way for innovative clinical interventions and achieve real clinical translation.

## AUTHOR CONTRIBUTIONS

Xudong Zhu, Guanliang Meng and Mila Jin designed this review. Lin Su, Jiawen Bu, Jiahui Yu, Mila Jin and Guanliang Meng retrieved relevant literatures. Lin Su, Xudong Zhu and Guanliang Meng wrote and revised this paper. All authors have read and approved the final manuscript.

## CONFLICT OF INTEREST STATEMENT

The authors declare that they have no competing interests.

## DATA AVAILABLE STATEMENT

The data and materials can be available from the corresponding author for rational reasons.

## ETHICS STATEMENT

Not applicable.
